# The Role of Immune Checkpoint Inhibitors in Cancer Therapy: Mechanism and Therapeutic Advances

**DOI:** 10.1002/mco2.70412

**Published:** 2025-10-05

**Authors:** Hengyi Chen, Hongling Yang, Lu Guo, Qingxiang Sun

**Affiliations:** ^1^ Department of Pulmonary and Critical Care Medicine The Seventh People's Hospital of Chongqing Chongqing China; ^2^ Department of Nephrology and Institute of Nephrology Sichuan Provincial People's Hospital School of Medicine Sichuan Clinical Research Centre for Kidney Diseases University of Electronic Science and Technology of China Chengdu China; ^3^ Department of Pulmonary and Critical Care Medicine Sichuan Provincial People's Hospital School of Medicine University of Electronic Science and Technology of China Chengdu China

**Keywords:** CTLA‐4, combination therapy, drug resistance, immune checkpoint inhibitors, PD‐1/PD‐L1, precision immunotherapy, tumor microenvironment

## Abstract

The rapid development of immune checkpoint inhibitors has fundamentally changed the landscape of cancer treatment. These agents restore T cell‐mediated antitumor immune responses by targeting key immune checkpoint molecules, thereby suppressing or eliminating tumors. However, their clinical application still faces multiple challenges, mainly including efficacy heterogeneity, drug resistance, immune‐related adverse events. Furthermore, there is still a lack of reliable biomarkers for predicting efficacy and toxicity. More critically, there is absence of precise predictive models that can systematically integrate multiomics features, dynamic tumor microenvironment evolution, and patient individual differences to comprehensively address the above issues. This review systematically summarizes the latest advancements in this field. The main contents include emerging targets like lymphocyte activation gene 3, T cell immunoreceptor with immunoglobulin and tyrosine‐based inhibitory motif domain, and mucin‐domain‐containing‐3, combination strategies, and the current research status and limitations of various predictive biomarkers. Moreover, it focuses on the potential of microbiome regulation, metabolic reprogramming, and artificial intelligence‐driven multiomics analysis technologies in achieving dynamic patient stratification and personalized treatment. By integrating the frontier research results and clinical insights, the review aims to provide a systematical theory framework and future directions for advancing precision immunotherapy.

## Graphical abstract

Immunotherapy, serving as a crucial breakthrough of precision medicine in the field of cancer treatment, among which immune checkpoint inhibitors (ICIs) have witnessed the most extensive application. This review systematically investigates the challenges, advancements, and future directions of ICIs in tumor treatment. These contents provide novel notions for the precise assessment and individualized treatment strategies of tumor patients undergoing ICI treatment, and contribute valuable insights to the transformation of ICIs from “broad‐spectrum treatment” to “precision immune intervention.”

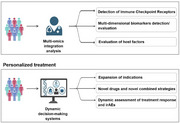

## 1 Introduction

Cancer remains the second leading cause of death globally [1]. Over the past decade, cancer therapy has undergone a paradigm shift driven by breakthroughs in immune checkpoint inhibitors (ICIs), which target molecules, such as cytotoxic T‐lymphocyte‐associated protein 4 (CTLA‐4), programmed death 1 (PD‐1)/programmed death‐ligand 1 (PD‐L1). By reversing T cell exhaustion and disrupting immunosuppressive networks within the tumor microenvironment (TME), ICIs have transformed treatment landscapes [2]. Since the approval of ipilimumab, the first CTLA‐4 inhibitor, for advanced melanoma in 2011 [3], PD‐1/PD‐L1 inhibitors have been increasingly adopted across various solid tumors, such as non‐small cell lung cancer (NSCLC), head and neck squamous cell carcinoma (HNSCC), and urothelial carcinoma, delivering durable remissions and long‐term survival for some patients. However, the objective response rates (ORRs) to ICI monotherapy vary significantly across tumor types [4, 5], with limited efficacy observed in certain solid tumors, such as microsatellite‐stable (MSS) colorectal cancer [6]. Clinical implementation of ICIs continues to face challenges, including drug resistance [7], immune‐related adverse events (irAEs) [8], as well as a lack of reliable biomarkers to guide personalized treatment decisions [9]. In this context, a systematic review of recent advancements, persistent challenges, and future directions in ICI treatment is essential for advancing precision cancer immunotherapy.

In recent years, emerging immune checkpoint molecules, such as lymphocyte activation gene (LAG)‐3, T cell immunoreceptor with immunoglobulin and tyrosine‐based inhibitory motif (ITIM) domain (TIGIT), and T cell immunoglobulin and mucin‐domain‐containing‐3 (TIM‐3), have garnered significant research interest [10]. Corresponding inhibitors and combination therapies targeting these pathways are under active investigation. Concurrent with advances in basic and clinical research, ICI‐based strategies are evolving from single‐target blockade to multitargeted, multidimensional precision interventions. Technological innovations such as single‐cell sequencing, spatial transcriptomics, and artificial intelligence (AI) have profoundly enhanced the understanding of tumor immune microenvironment (TIME) heterogeneity and its dynamic evolution [11, 12]. Meanwhile, metabolic modulation and epigenetic regulation offer promising approaches to reshape the TIME and improve responsiveness to ICIs. The development of new biomarkers, coupled with multiomics integration and AI‐driven predictive models, holds great potential for establishing personalized treatment frameworks and enabling dynamic precision oncology.

While ICIs have revolutionized cancer treatment, their clinical translation faces critical bottlenecks. First, the predictive power of established biomarkers like PD‐L1 expression and tumor mutaional burden (TMB) remains limited, with accuracy heavily compromised by tumor heterogeneity [13, 14, 15]. Second, the molecular mechanisms underlying irAEs are incompletely understood [16], and effective predictive or preventive strategies are lacking. Additionally, the absence of standardized guidelines for optimizing combination therapy administration sequences and dosages [17] introduces uncertainty and complexity into clinical practice. To address these challenges, future research must integrate cutting‐edge advances from immunology, computational biology, and clinical medicine to drive a paradigm shift in tumor immunotherapy from broad‐spectrum approaches toward precise, individualized treatment models.

This review comprehensively examines the mechanisms of action and emerging targets of ICIs, while evaluating recent advances in combination strategies with chemotherapy, antiangiogenic agents, radiotherapy, and other modalities. It critically analyzes current bottlenecks and challenges, including identifying responsive patient populations, understanding resistance mechanisms, predicting and assessing treatment efficacy, and managing irAEs. A key focus is placed on advancements in biomarker discovery through multiomics approaches and AI‐driven precision prediction models, which aim to optimize therapeutic decision‐making. Looking ahead, the review outlines a transformative pathway for precision immunotherapy, emphasizing dynamic patient stratification, individualized interventions, and multitechnology integration. By providing a structured theoretical framework, this work seeks to guide future research and clinical development in the field.

## 2 Immune Checkpoints and Blocking Strategies

**FIGURE 1:**
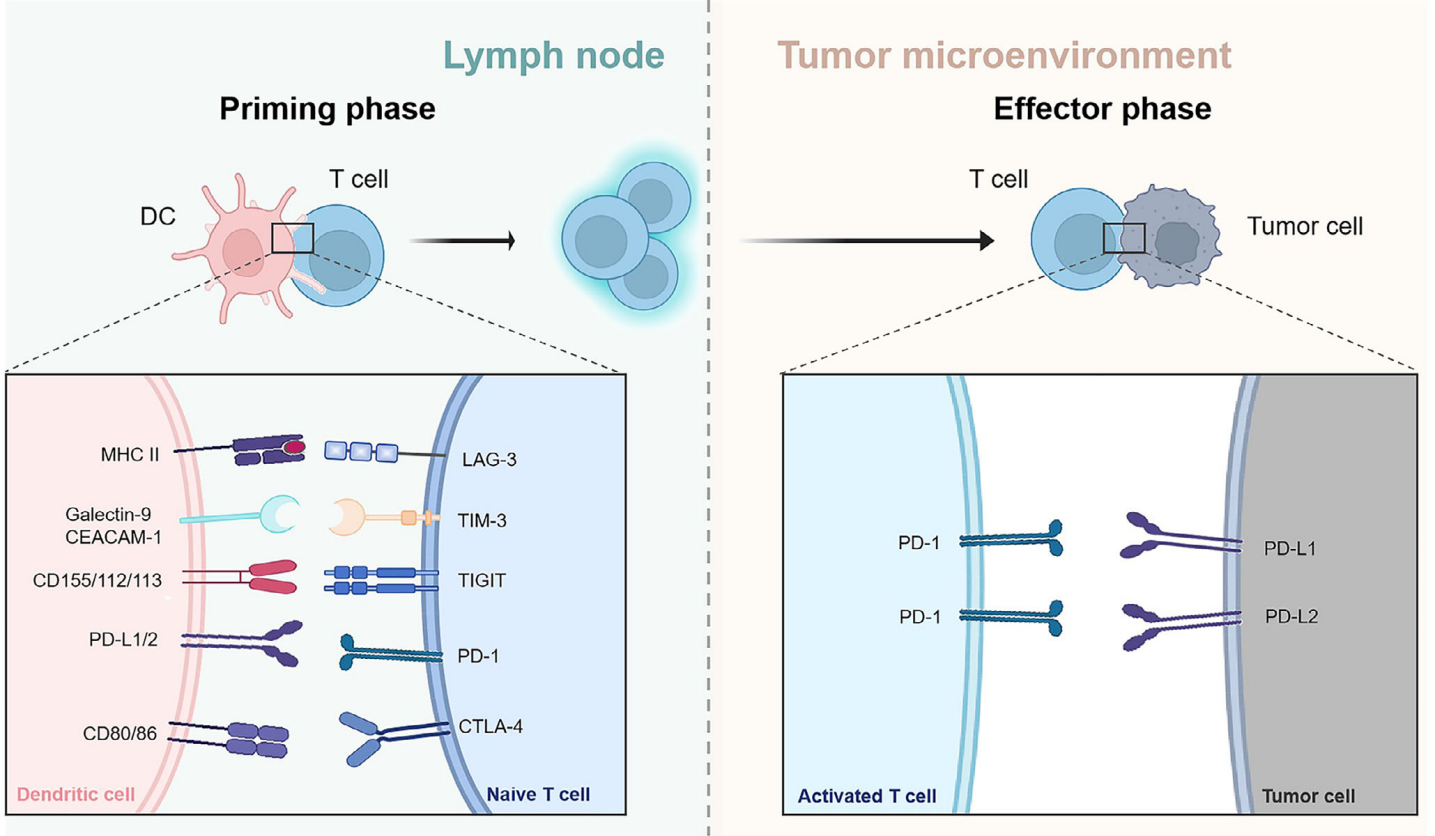
The distribution characteristics of common inhibitory immune checkpoint molecules in the activation (lymph nodes) and effector (TME) stages of T cells. In the priming phase, DCs activate naive T cells through antigen presentation, and inhibitory receptors such as LAG‐3, TIM‐3, TIGIT, PD‐1, and CTLA‐4 on the surface of naive T cells can negatively regulate the activation process of T cells by binding to their corresponding ligands. In the effector phase, activated effector T cells interact with PD‐L1/L2 on the surface of tumor cells through PD‐1, mediating the phenomenon of tumor immune escape; while persistent inhibitory signals may result in T cell exhaustion. DC, dendritic cell; MHC, major histocompatibility complex; CEACAM‐1, carcinoembryonic antigen‐related cell adhesion molecule 1; PD‐L1/2, programmed cell death‐ligand 1/2; LAG‐3, lymphocyte activation gene 3; TIM‐3, T cell immunoglobulin and mucin‐domain‐containing‐3; TIGIT, T cell immunoreceptor with immunoglobulin and tyrosine‐based inhibitory motif (ITIM) domain; PD‐1, programmed cell death 1; CTLA‐4, cytotoxic T‐lymphocyte‐associated antigen‐4.

Immune checkpoint molecules, encompassing both inhibitory and stimulatory subtypes, are essential for maintaining immune homeostasis by regulating T cell activity and preventing autoimmune tissue damage [18]. Tumor cells frequently hijack epigenetic modifications and signaling pathways to upregulate coinhibitory immune checkpoint molecules, such as PD‐1, CTLA‐4, LAG‐3, TIGIT, and TIM‐3, and their ligands (Figure 1). This suppression of tumor‐infiltrating lymphocytes (TILs) enables immune evasion and drives tumor progression [19].

ICIs specifically disrupt interactions between inhibitory checkpoints on tumor or immune cells and their ligands, restoring and enhancing effector T cell activation, proliferation, and cytotoxicity. Moreover, it promotes T cell infiltration into the TME, facilitating tumor cell recognition and elimination to exert antitumor effects [20]. A thorough summary of immune checkpoints and their corresponding therapies is provided in Table 1.

**TABLE 1:** Monoclonal antibodies targeting immune checkpoints with regulatory approval as of August 2025.

Target	Agents	First approval	Main approved indications
PD‐1	Pembrolizumab (KEYTRUDA)	US FDA (2014)	NSCLC, melanoma, HNSCC, classical Hodgkin lymphoma, PMBCL, bladder cancer, esophageal cancer, gastric cancer, colorectal cancer, cervical cancer, HCC, biliary tract cancer, TNBC, urothelial carcinoma, Merkel cell carcinoma, RCC, endometrial cancer, CSCC, MPM, basal cell carcinoma
	Nivolumab (OPDIVO)	PMDA (2014)	NSCLC, melanoma, HNSCC, classical Hodgkin lymphoma, bladder cancer, esophageal cancer, gastric cancer, colorectal cancer, HCC, urothelial carcinoma, renal cell carcinoma, MPM
	Cemiplimab (LIBTAYO)	US FDA (2018)	CSCC, NSCLC, basal cell carcinoma
	Toripalimab (TUOYI)	NMPA (2018)	Melanoma, nasopharyngeal carcinoma, urothelial carcinoma, ESCC, NSCLC
	Sintilimab (TYVYT)	NMPA (2018)	Hodgkin's lymphoma, NSCLC, hepatocellular carcinoma, ESCC, gastric adenocarcinoma, endometrial cancer, RCC
	Camrelizumab (AiRuiKa)	NMPA (2019)	Hodgkin's lymphoma, HCC, NSCLC, nasopharyngeal carcinoma, ESCC, gastric/gastroesophageal junction cancer
	Tislelizumab (TEVIMBRA)	NMPA (2019)	ESCC, gastric adenocarcinoma, adenocarcinoma of the gastroesophageal junction, NSCLC
	Prolgolimab	Minzdrav‐RF (2020)	Melanoma
	Dostarlimab (JEMPERLI)	US FDA (2021)	Cervical cancer
	Penpulimab (Anike)	NMPA (2021)	Nasopharyngeal carcinoma, Hodgkin's lymphoma, NSCLC
	Serplulimab (Hetronifly)	NMPA (2022)	Extensive‐stage SCLC
	Finotonlimab (Anyouping)	NMPA (2025)	Head and neck squamous cell carcinoma
	Retifanlimab (ZYNYZ)	US FDA (2025)	Merkel cell carcinoma
PD‐L1	Atezolizumab (TECENTRIQ)	US FDA (2016)	NoSCLC, SCLC, TNBC, HCC, melanoma, ASPS, urothelial carcinoma
	Durvalumab (IMFINZI)	US FDA (2017)	NSCLC, SCLC, HCC, biliary tract cancer, endometrial cancer
	Avelumab (BAVENCIO)	US FDA (2017)	Merkel cell carcinoma, urothelial carcinoma, RCC
	Envafolimab (ENWEIDA)	NMPA (2021)	MSI‐H or dMMR advanced solid tumors
	Sugemalimab (Cejemly)	NMPA (2021)	NSCLC, lymphoma, gastric/gastroesophageal junction adenocarcinoma
	Cosibelimab (UNLOXCYT)	US FDA (2024)	Squamous cell carcinoma of the skin
	Tagitanlimab (KL‐A167)	NMPA (2024)	Nasopharyngeal carcinoma
CTLA4	Ipilimumab (YERVOY)	US FDA (2011)	Melanoma, RCC, MSI‐H colorectal cancer, lung cancer, HCC
	Tremelimumab (IMJUDO)	US FDA (2022)	HCC, lung cancer
LAG‐3	Relatlimab (OPDUALAG)	US FDA (2022)	Melanoma

*Data sources*: Drugs@FDA (https://www.accessdata.fda.gov/scripts/cder/daf/index.cfm), NMPA (https://www.nmpa.gov.cn/datasearch/home‐index.html), PMDA (https://www.pmda.go.jp/) and GRLS (https://grls.rosminzdrav.ru/).

Abbreviations: ASPS, alveolar soft part sarcoma; CSCC, cutaneous squamous cell carcinoma; CTLA4, cytotoxic T‐lymphocyte‐associated protein 4; dMMR, deficient mismatch repair; ESCC, esophageal squamous cell carcinoma; US FDA, United States Food and Drug Administration; GRLS, State Register of Medicines; HCC, hepatocellular carcinoma; HNSCC, head and neck squamous cell carcinoma; LAG‐3, lymphocyte activation gene 3; Minzdrav‐RF, Ministry of Health of the Russian Federation; MPM, malignant pleural mesothelioma; MSI‐H, microsatellite instability‐high; NMPA, National Medical Products Administration; NSCLC, non‐small cell lung cancer; PD‐1, programmed cell death protein 1; PD‐L1, programmed death‐ligand 1; PMBCL, primary mediastinal large B‐cell lymphoma; PMDA, Pharmaceuticals and Medical Devices Agency; RCC, renal cell carcinoma; SCLC, small‐cell lung cancer; TNBC, triple‐negative breast cancer.

### 2.1 The PD‐1/PD‐L1 Pathway

**FIGURE 2:**
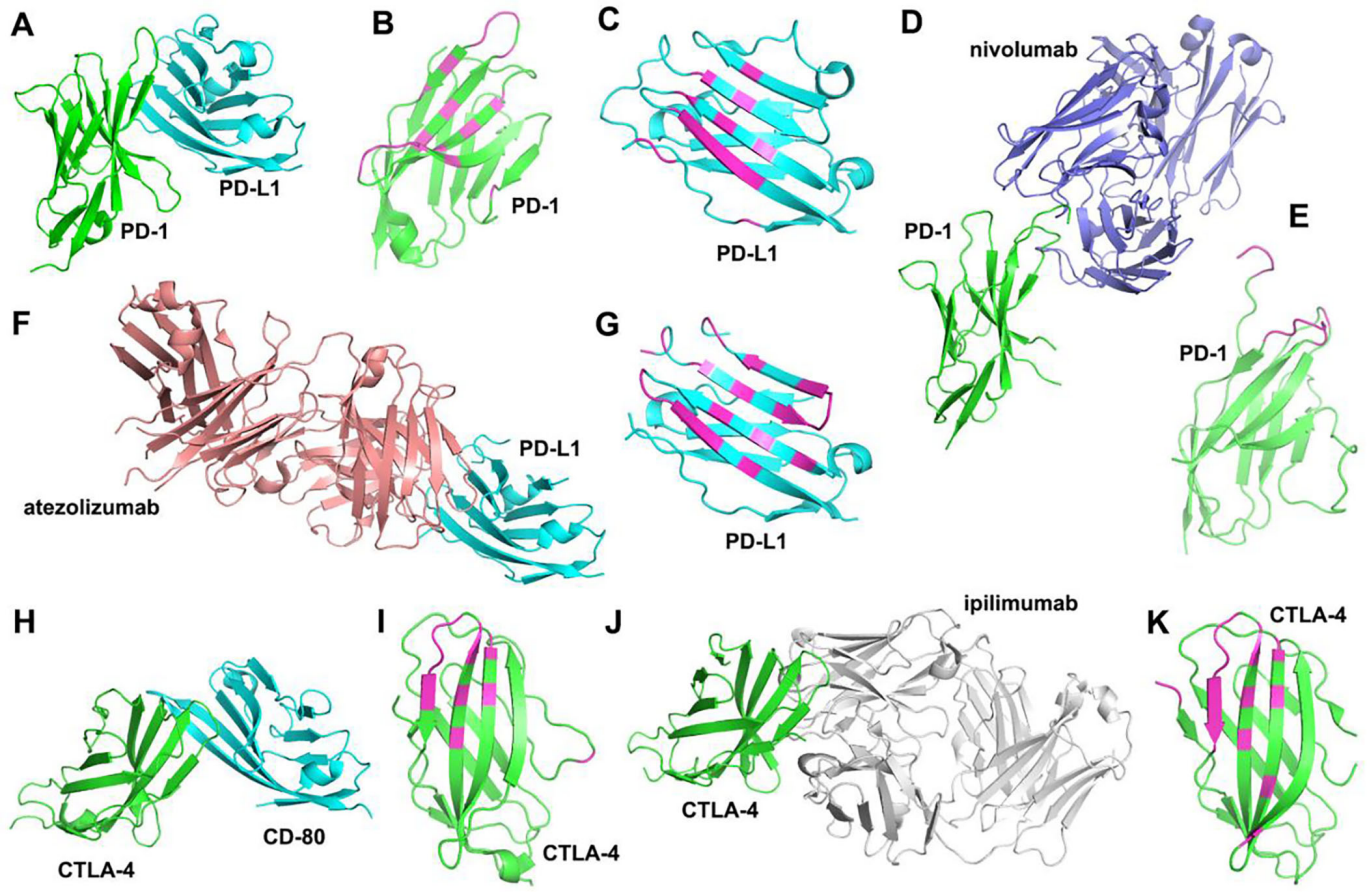
Inhibition mechanism of ICIs. (A) Complex structure of PD‐1 and PD‐L1 (3BIK) shown in cartoon representation. (B) The PD‐1 structure in panel A with the PD‐L1 interacting residues (within 4 Å distance) colored in magenta. (C) The PD‐L1 structure in panel A, with the PD‐1 interacting residues colored in magenta. (D) Complex structure of PD‐1 and nivolumab (5GGR). (E) The PD‐1 structure in panel D with the nivolumab interacting residues colored in magenta. (F) Complex structure of PD‐L1 and atezolizumab (5XXY). (G) The PD‐L1 structure in panel F, with the atezolizumab interacting residues colored in magenta. (H) Complex structure of CTLA‐4 and CD80 (1I8L). (I) The CTLA‐4 structure in panel H, with the CD80‐interacting residues colored in magenta. (J) Complex structure of CTLA‐4 and ipilimumab (5TRU). (K) The CTLA‐4 structure in panel J with the ipilimumab interacting residues colored in magenta.

The PD‐1/PD‐L1 axis represents a fundamental mechanism enabling tumor immune evasion. PD‐1, an immunosuppressive receptor, is primarily expressed on activated T cells, regulatory T cells (Tregs), and exhausted T cells, while its ligand PD‐L1 is commonly upregulated on tumor cells, antigen‐presenting cells (APCs), including dendritic cells (DCs) and macrophages, and other stromal components within the TME. Structurally, both PD‐1 and PD‐L1 feature an extracellular immunoglobulin variable (IgV)‐like domain that facilitates high‐affinity binding (Figure 2A). PD‐1 primarily interacts with PD‐L1 through two loops and four beta strands, engaging four beta strands in PD‐L1 (Figure 2B,C). Upon binding, PD‐1 recruits phosphatases through Src homology region 2 domain‐containing phosphatase‐2, thereby inhibiting downstream T cell receptor (TCR) signaling pathways, such as phosphatidylinositol 3‐kinase (PI3K)/protein kinase B (PKB/AKT) and rat sarcoma viral oncogene homologue/extracellular signal‐regulated kinase (ERK) kinase (MEK)/ERK pathways, and this suppression reduces T cell activation, proliferation, and effector functions, ultimately resulting in T cell exhaustion [21]. Functionally exhausted T cells exhibit diminished secretion of effector cytokines, such as interferon (IFN)‐γ, tumor necrosis factor (TNF)‐α, and interleukin (IL)‐2, diminished proliferative capacity, and impaired cytotoxic activity [22].

Targeting the PD‐1/PD‐L1 pathway has become a cornerstone of cancer immunotherapy. Monoclonal antibodies that block PD‐1/PD‐L1 interactions disrupt this immunosuppressive mechanism, thereby reinvigorating T cell‐mediated antitumor immunity. For instance, the anti‐PD‐1 antibody nivolumab binds directly to three loops of PD‐1 that partially overlap with the PD‐L1 binding interface (Figure 2D,E), while the anti‐PD‐L1 agent atezolizumab sterically blocks PD‐1 engagement by occupying its binding site on PD‐L1 (Figure 2F,G). These mechanistic insights have driven clinical advancements: anti‐PD‐1/PD‐L1 antibodies are now standard treatments for multiple malignancies, including melanoma, NSCLC, renal cell carcinoma (RCC), and classical Hodgkin's lymphoma [23].

### 2.2 The CTLA‐4 Pathway

T cell activation relies on dual signals: TCR recognition of peptide–major histocompatibility complex (MHC) complexes and a costimulatory signal delivered through CD28 binding to B7 family ligands (CD80/CD86) [24]. This interaction drives T cell proliferation, IL‐2 secretion, and expression of survival genes, such as Bcl‐2 family members, via PI3K/AKT and nuclear factor‐kappaB (NF‐κB) pathway activation, enabling clonal expansion and effector differentiation [25]. CTLA‐4, a high‐affinity inhibitory receptor, is upregulated on activated effector T cells and constitutively expressed on Tregs. Like PD‐1/PD‐L1, CTLA‐4 uses its IgV domain to bind CD80/CD86 (Figure 2H,I), competitively blocking CD28 engagement and inhibiting T cell proliferation through “ligand sequestration” [26]. On Tregs, constitutive CTLA‐4 expression further suppresses immunity by promoting endocytic degradation of B7 molecules, thereby depleting costimulatory ligands for CD28 [27]. CTLA‐4 indirectly dampens antitumor responses by stimulating the secretion of immunosuppressive cytokines such as TGF‐β and IL‐10 [28].

CTLA‐4 inhibitors, such as the monoclonal antibody ipilimumab, restore T cell function by blocking CTLA‐4/B7 interactions. Ipilimumab binds to the IgV domain of CTLA‐4, sterically preventing CD80/CD86 engagement (Figure 2J,K), thereby lifting T cell suppression and attenuating the activity of Tregs. As the first ICI approved for clinical use, ipilimumab is indicated for malignancies including melanoma, RCC, and hepatocellular carcinoma (HCC) [29]. Nevertheless, its efficacy is often limited by tumor heterogeneity, immunosuppressive metabolic conditions, and significant irAEs. Compared with PD‐1/PD‐L1 inhibitors, CTLA‐4 inhibitors have a narrower therapeutic scope and are predominantly used in combination strategies. Combining CTLA‐4 inhibitors, such as ipilimumab and tremelimumab, with PD‐1/PD‐L1 inhibitors enhances response rates and survival outcomes in melanoma and NSCLC, as demonstrated in trials like POSEIDON [30, 31, 32, 33]. Expanding the combinatorial arsenal, novel agents such as cadonilimab—a bispecific antibody targeting both PD‐1 and CTLA‐4—have received approval in China for cervical cancer treatment [34]. These developments highlight a shift toward multitarget regimens, biomarker‐driven therapy, and rationally designed combinations to broaden clinical utility.

### 2.3 Novel Immune Checkpoints and Novel ICIs

Beyond the well‐established PD‐1/PD‐L1 and CTLA‐4 pathways, emerging immune checkpoints like LAG‐3, TIGIT, TIM‐3, V‐domain Ig suppressor of T‐cell activation (VISTA), and B‐ and T‐lymphocyte attenuator (BTLA) are attracting considerable interest as promising targets in tumor immunotherapy. These molecules promote tumor immune escape through diverse mechanisms, making them compelling candidates for combination therapy approaches aimed at overcoming resistance and enhancing treatment efficacy.

#### 2.3.1 Lymphocyte Activation Gene 3

LAG‐3 is expressed on activated T cells, natural killer (NK) cells, and Tregs, where it modulates antitumor immunity through multiple mechanisms. Primarily, LAG‐3 interacts with MHC class II molecules on APCs or binds to fibrinogen‐like protein 1 secreted by tumor cells [35]. This engagement disrupts TCR/MHC‐II signaling and suppresses IL‐2 and IFN‐γ production, thereby inhibiting both CD4+ and CD8+ T cell activation [36]. The intracellular domain of LAG3 recruits the E3 ubiquitin ligase casitas B‐lineage lymphoma proto‐oncogene‐b (Cbl‐b), promoting degradation of downstream TCR signaling components and often acting synergistically with PD‐1 to drive T cell exhaustion [37]. Highly expressed on Tregs, LAG‐3 reinforces an immunosuppressive TME by suppressing immune cell activation, promoting Treg differentiation through TGF‐β and IL‐10 secretion [38], and facilitating M2‐like polarization of tumor‐associated macrophages (TAMs) [39], further enhancing immune evasion.

#### 2.3.2 T Cell Immunoreceptor with Immunoglobulin and ITIM Domain

TIGIT is expressed on NK cells, T cells, and DCs, where it facilitates tumor immune evasion through ligand competition, inhibition of activating signaling pathways, and remodeling of the TIME. A key mechanism involves TIGIT competitively binding to CD155 and CD112 on tumor cells, thereby blocking their interaction with the costimulatory receptor CD226 [40]. This competition dampens the cytotoxic activity of NK cells and T cells while paradoxically enhancing IFN‐γ secretion in some contexts. Intracellularly, ITIM, the intracellular domain of TIGIT, recruits SH2‐containing inositol phosphatase 1, which attenuates PI3K/AKT and MAPK signaling [41], resulting in metabolic reprogramming of T cells, such as impaired glucose uptake and reduced proliferation. In addition, it also inhibits NF‐κB signaling, diminishing production of critical effector cytokines including IL‐2 and TNF‐α, and further impairing CD8+ T cell function within the TME [42]. Highly expressed on Tregs, TIGIT reinforces immune suppression by modulating DCs’ function through IL‐10 and TGF‐β secretion [43] and promoting M2‐like polarization of TAMs [44, 45]. Elevated TIGIT expression is strongly associated with T cell exhaustion, as it synergizes with PD‐1 to inhibit CD226‐mediated costimulation and PI3K pathway activation, driving dysfunctional T cell states [46].

#### 2.3.3 T Cell Immunoglobulin and Mucin‐Domain‐Containing‐3

TIM‐3, a member of the TIM protein family, is broadly expressed on activated CD8+ T cells, Tregs, NK cells, DCs, TAMs, and malignant cells across multiple cancer types, such as melanoma, NSCLC, prostate cancer, colorectal carcinoma, and HCC [47]. By binding its ligands galectin‐9 and high mobility group box 1 protein, TIM‐3 exerts immunosuppressive effects through multiple mechanisms: it dampens Th1/Th17 responses [48], drives CD8+ T cell exhaustion [49], enhances Treg activity [47], reduces secretion of IFN‐γ and TNF‐α [50], and attenuates DC‐dependent innate immunity [51]. Within the TME, TIM‐3‐high Tregs further suppress antitumor immunity through TGF‐β and IL‐10 secretion [52]. TIM‐3 frequently coexpresses with PD‐1, synergistically impairing T cell function by dual inhibition of TCR signaling and cytokine production, such as IL‐2 and IFN‐γ, leading to diminished proliferation and cytotoxicity [53]. In cells coexpressing both receptors, TIM‐3 additionally regulates Treg function to further blunt immune responses [54]. Furthermore, compensatory TIM‐3 upregulation following PD‐1/PD‐L1 blockade serves as an adaptive resistance mechanism, enabling tumor escape through alternative inhibitory signaling [55]. Emerging evidence also highlights posttranslational regulation of TIM‐3, such as palmitoylation at cysteine 296 by the acyltransferase DHHC9, which enhances TIM‐3 stability by preventing E3 ubiquitin ligase HRD1‐mediated degradation [56].

#### 2.3.4 Novel ICIs

In recent years, inhibitors targeting emerging immune checkpoints such as LAG‐3, TIGIT, and TIM‐3 have shown promise in overcoming resistance to PD‐1/CTLA‐4 inhibitors.

LAG‐3 inhibitors, exemplified by relatlimab, restore T cell activity by blocking the interaction between LAG‐3 and MHC‐II, demonstrating synergistic antitumor efficacy in solid tumors such as melanoma [57] and NSCLC [58]. Relatlimab, approved in combination with nivolumab for melanoma, has paved the way for other agents such as LBL‐007, a fully human monoclonal antibody that enhances T cell activation and IL‐2 secretion [59]. In a phase II trial, LBL‐007 combined with the PD‐1 inhibitor toripalimab achieved an ORR of 33.3%, a disease control rate (DCR) of 75%, and a median progression‐free survival (PFS) of 10.8 months in nasopharyngeal carcinoma [60], underscoring the potential of this combination strategy.

The TIGIT inhibitor tiragolumab, evaluated in the phase II CITYSCAPE trial alongside atezolizumab, improved outcomes in PD‐L1‐high NSCLC, increasing ORR from 16.2 to 31.3% and extending median PFS from 3.6 to 5.4 months (NCT03563716) [61]. Mechanistic studies suggest that dual TIGIT and PD‐1/PD‐L1 blockade may synergistically enhance antitumor immunity, potentially through NK‐cell‐mediated activation of CD8+ TILs [62]. These findings position TIGIT as a key target for next‐generation immunotherapy. However, the TIGIT inhibitor vibostolimab, when combined with pembrolizumab, failed to meet the primary overall survival (OS) endpoint in an SCLC trial (NCT05224141), highlighting the need for refined patient selection. Current research focuses on developing bispecific antibodies, optimizing combination strategies, and identifying predictive biomarkers to maximize TIGIT inhibition's clinical benefits.

Preclinical studies demonstrate that TIM‐3 inhibition can remodel the immunosuppressive TME by enhancing CD8+ T and NK cell infiltration and restoring their functionality [63]. A range of TIM‐3‐targeting strategies is under investigation, including monoclonal antibodies, bispecific agents, and small‐molecule peptides. While the phase III trial of the TIM‐3 inhibitor sabatolimab in patients with moderate, high, or extremely high‐risk myelodysplastic syndrome (MDS) or chronic myelomonocytic leukemia‐2 failed to meet its primary OS endpoint [64], combinations of sabatolimab with hypomethylating agents or the anti‐PD‐1 antibody spartalizumab have shown acceptable safety and preliminary efficacy in high‐risk MDS [65] and advanced solid tumors [66]. Bispecific antibodies targeting TIM‐3 alongside another immune checkpoint, such as PD‐L1, represent a promising multitarget approach, though molecules such as LY3415244 have faced challenges related to immunogenicity [67]. Future development of TIM‐3 inhibitors will likely require biomarker‐driven strategies, such as the assessment of palmitoylation status of TIM‐3 [56], and innovative bispecific designs to enhance therapeutic efficacy.

Overall, novel ICIs offer significant potential to expand the cancer immunotherapy toolkit. By leveraging multitarget inhibition, TME reprogramming, and immune profiling‐based patient stratification, these agents may advance the field toward more precise and personalized treatment paradigms.

## 3 Therapeutic Advances in ICIs

**FIGURE 3:**
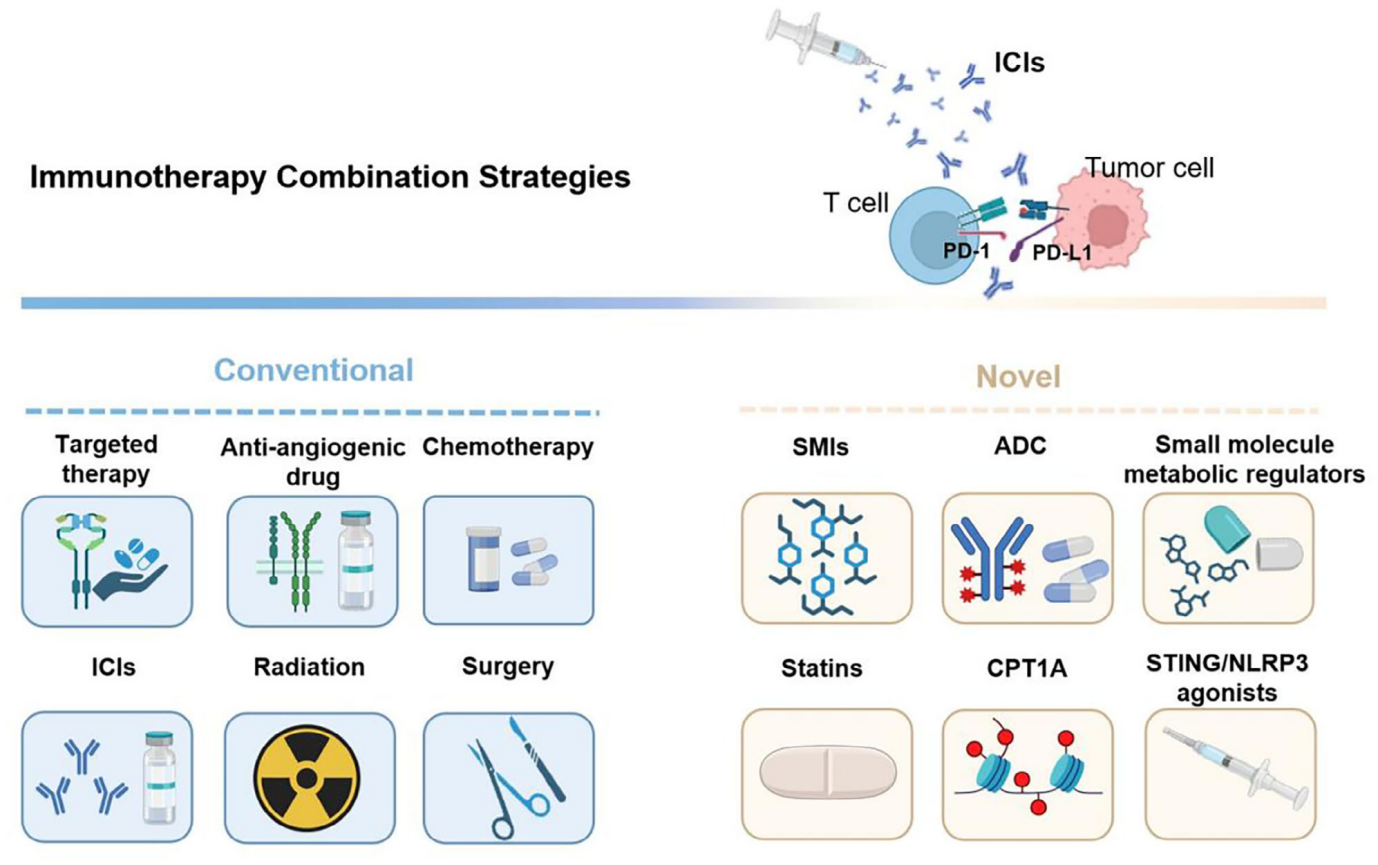
Immunotherapy combination strategies: an overview of conventional and novel approaches. Common combination strategies for immunotherapy encompass targeted therapy, antiangiogenic agents, chemotherapy, ICIs, radiotherapy, and surgery. These are currently the widely adopted combination modalities of immunotherapy in clinical practice. Innovative combination strategies for immunotherapy include SMIs, ADCs, small molecule metabolic regulators, statins, CPT1A‐related formulations, STING/NLRP3 agonists, and so on. These strategies represent the forefront directions of exploration in immunotherapy combination strategies. ICIs, immune checkpoint inhibitors; SMIs, small molecule immunomodulators; ADC, antibody–drug conjugates; CPT1A, carnitine palmitoyltransferase 1A; STING, stimulator of interferon genes; NLRP3, nod‐like receptor protein 3.

ICIs have revolutionized oncology treatment paradigms, transitioning from standalone monotherapy to sophisticated multimodal combinations that have substantially improved survival outcomes across diverse tumor types. Current clinical exploration predominantly focuses on PD‐1/PD‐L1 inhibitor‐based regimens combined with targeted therapies, chemotherapy, radiotherapy, and other modalities (Figure 3). These strategies synergistically amplify antitumor immune responses, representing a pivotal research frontier in immuno‐oncology.

### 3.1 Advancement in Monotherapy with ICIs

ICI monotherapy continues to demonstrate meaningful clinical benefit in specific tumor subtypes with well‐defined molecular and immune profiles. In advanced melanoma, ICIs serve as a cornerstone of treatment [68], achieving 5‐year survival rates exceeding 30% in select cohorts, with particularly robust responses observed in patients with high TMB [69]. Certain rare sarcomas, such as alveolar soft part sarcoma (ASPS), also exhibit marked sensitivity to PD‐1 inhibitors, likely due to a highly immune‐infiltrated TIME and the presence of fusion genes like ASPSCR1–TFE3 [70, 71]. In RCC, nivolumab monotherapy as second‐line treatment extends median OS to 25 months, outperforming historical outcomes with targeted therapies [72]. Similarly, patients with previously treated microsatellite instability‐high (MSI‐H) or deficient mismatch repair (dMMR) colorectal cancer derive substantial benefit from pembrolizumab, achieving an ORR of 34.9%, durable survival, and manageable toxicity [73]. Durable responses are also reported in subsets of NSCLC, bladder cancer, and advanced esophageal cancer with high PD‐L1 expression [74, 75, 76]. However, ICI monotherapy shows limited efficacy in patients with MSS or proficient DNA mismatch repair status, where ORRs typically remain below 5% [77]. These findings highlight the critical importance of biomarker‐driven patient selection to optimize outcomes and inform the development of rational combination strategies.

### 3.2 Emerging Immunotherapy Combination Strategies

Combination immunotherapy has emerged as a pivotal strategy to address limitations in current cancer treatments, harnessing synergistic mechanisms to amplify antitumor immune responses. Its primary applications include dual ICI regimens and the integration of ICIs with other antitumor modalities.

#### 3.2.1 Immunotherapy Combination Strategy

Dual immune checkpoint blockade, particularly the cotargeting of PD‐1 and CTLA‐4, has demonstrated improved ORRs and long‐term survival in malignancies such as melanoma, RCC, NSCLC, and HCC. However, this approach is frequently associated with a higher incidence of irAEs [78, 79, 80, 81]. Emerging dual blockade strategies, such as combining LAG‐3 and PD‐1 inhibitors (e.g., relatlimab plus nivolumab), have demonstrated promising efficacy with acceptable safety profiles. In melanoma, this regimen extended median PFS to 10.1 months (more than double that of anti‐PD‐1 monotherapy), while maintaining a manageable rate of grade 3–4 irAEs (18.9%), offering a viable option for traditionally immunotherapy‐resistant “cold tumors” [82]. In addition, a phase III trial evaluating the anti‐TIGIT antibody BGB‐A1217 in combination with the PD‐1 inhibitor tislelizumab for first‐line treatment of NSCLC and esophageal squamous cell carcinoma (ESCC) is underway, with results poised to potentially reshape standard‐of‐care approaches for these tumors.

Combining ICIs with tumor vaccines has emerged as a promising strategy with strong therapeutic synergy. For instance, mRNA neoantigen vaccines combined with pembrolizumab significantly extended recurrence‐free survival in melanoma patients without increasing adverse events [83]. Similarly, in relapsed/refractory large B‐cell lymphomas, the combination of CD19 chimeric antigen receptor T‐cell therapy and pembrolizumab achieved a clinical benefit rate of up to 33% with favorable tolerability [84]. Oncolytic viruses, such as talimogene laherparepvec, combined with pembrolizumab, enhanced CD8+ T cell infiltration and PD‐L1 upregulation in both injected and distant lesions, achieving a 62% response rate in melanoma [85]. However, not all combinations have proven successful. Despite the good tolerability of the indoleamine 2,3‐dioxygenase (IDO) inhibitor (epacadostat) and PD‐1 blockade therapy (pembrolizumab), it failed to improve outcomes in either NSCLC [86] or unresectable/metastatic melanoma [87], highlighting the challenges of translating mechanistic synergy into clinical efficacy.

These findings underscore the importance of developing predictive biomarkers and patient stratification strategies to guide the rational selection of combination therapies. Precision in patient selection and thoughtfully designed, layered regimens will be critical to maximizing therapeutic benefits while maintaining safety.

#### 3.2.2 ICIs Combined with Targeted Therapy

The combination of ICIs and targeted therapies has emerged as a transformative approach for treating various solid tumors, leveraging synergistic effects to reshape the TME and amplify antitumor immune responses. Targeted agents can enhance the immune‐permissive TIME through several mechanisms. For example, epidermal growth factor receptor (EGFR)‐tyrosine kinase inhibitors (TKIs) suppress the MEK/ERK pathway, upregulating MHC‐I expression to improve tumor antigen presentation, promoting CD8+ T cell infiltration, and inducing PD‐L1 expression [88]. In mesenchymal–epithelial transition factor (MET)‐amplified tumors, MET inhibitors block the hepatocyte growth factor/MET axis, reducing M2‐type macrophage polarization and fostering a TME more conducive to CD8+ T cell recruitment [89]. In ovarian cancer models, the poly(ADP‐ribose) polymerase inhibitor olaparib induces DC maturation and T cell activation, and exhibits a synergistic effect with anti‐PD‐1 therapy [90].

Notably, resistance to targeted therapies often coincides with heightened immune evasion mechanisms. For example, in NSCLC cell lines, the anaplastic lymphoma kinase (ALK) fusion protein upregulates PD‐L1 expression, impairing T cell function [91]. Consistently, patients who develop resistance to ALK‐TKIs exhibit elevated PD‐L1 levels, with case reports documenting complete clinical remission following subsequent treatment with ICI [92].

In untreated, advanced biliary tract cancer, the global phase III TOPAZ‐1 trial established durvalumab combined with gemcitabine and cisplatin as a new first‐line standard, achieving a median OS of 12.9 months (95% CI 11.6–14.1) without new safety concerns [93]. Building on this regimen, a triplet combination of durvalumab, hepatic artery infusion chemotherapy, and lenvatinib (antivascular endothelial growth factor receptor [VEGFR]/MET) demonstrated an ORR of 48.4% and extended median OS to 17.7 months in selected patients, suggesting added clinical benefit [94]. Similarly, in human EGFR 2 (HER2)‐positive gastric cancer patients with PD‐L1 CPS ≥1, a phase II trial evaluating trastuzumab deruxtecan alongside fluorouracil plus cisplatin and pembrolizumab reported an ORR of 58%, indicating robust antitumor activity. However, this regimen was associated with a high rate of grade ≥3 treatment‐related adverse events (91%) [95], raising tolerability concerns that may limit its broader applicability. In treatment‐naïve advanced NSCLC with EGFR‐sensitizing mutations, combinations of ICIs and EGFR‐TKIs achieved ORRs ranging from 41.7 to 43% [96, 97]. Yet these regimens offered only modest efficacy improvements over TKI monotherapy while introducing significantly higher toxicity, underscoring persistent safety challenges.

Overall, the clinical outcomes of combining ICIs with targeted therapies remain heterogeneous. While many targeted agents modulate immune cell function, providing a strong rationale for synergistic activity, the US FDA has approved at least six such regimens across various malignancies. However, these strategies are a “double‐edged sword,” requiring careful consideration of efficacy‐toxicity trade‐offs. Their long‐term safety and durability of response continue to warrant validation in larger prospective studies.

#### 3.2.3 ICIs Combined with Antiangiogenic Agents

Antiangiogenic agents, including monoclonal antibodies or small‐molecule TKIs targeting the vascular endothelial growth factor (VEGF)–VEGFR pathway, have become a pivotal strategy for enhancing ICI efficacy. VEGF‐mediated immunosuppression impairs T cell inhibition, promotes the recruitment of Tregs and myeloid‐derived suppressor cells (MDSCs), as well as inhibits DC maturation [98]. By blocking VEGF, these agents not only counteract these immunosuppressive effects but also normalize tumor vasculature, facilitating CD8+ T cell infiltration and synergizing with ICIs [99].

Multiple phase III trials have established the combination of antiangiogenic agents and ICIs as a standard first‐line therapy for advanced RCC, demonstrating significant improvements in PFS and/or OS [100, 101, 102]. In HCC, two phase III trials compared ICI–antiangiogenic combinations (atezolizumab combined with bevacizumab [103] and cabozantinib combined with atezolizumab [104]) with sorafenib, both yielding positive results.

However, the benefits of these combinations are not universal. In the JAVELIN Renal 101 trial, avelumab plus axitinib improved PFS but did not significantly extend OS in RCC [105]. Similarly, in the COSMIC‐312 trial, the OS benefit of atezolizumab combined with cabozantinib in HCC did not surpass that of sorafenib [106]. The LEAP‐002 trial also failed to confirm an OS advantage with pembrolizumab plus lenvatinib in HCC [107], and in ovarian cancer, the IMagyn050 trial showed no significant OS improvement with atezolizumab plus bevacizumab and chemotherapy [108]. These findings highlight the tumor‐type‐specific variability in response to ICI and antiangiogenic combination therapies, emphasizing the need for tailored approaches in different malignancies.

#### 3.2.4 ICIs Combined with Chemotherapy

The combination of ICIs and chemotherapy marks a significant breakthrough in oncology. Chemotherapeutic agents can induce immunogenic cell death, promoting the release of tumor antigens and enhancing their presentation [109]. Additionally, chemotherapy may activate tumor‐specific T cells while depleting immunosuppressive cells such as Tregs and MDSCs [110], thereby reshaping the TIME. These mechanisms underpin the synergistic antitumor effects observed with ICIs and may help overcome resistance due to immune evasion.

Clinical trials across various solid tumors have consistently shown the superiority of ICI–chemotherapy combinations over chemotherapy alone. For instance, in advanced NSCLC, combining PD‐1/PD‐L1 inhibitors with platinum‐based chemotherapy significantly improves the ORR, OS, and PFS [111, 112, 113]. Similar improvements in ORR and PFS have been observed in gastric cancer [114] and urothelial carcinoma [115], with promising activity also reported in triple‐negative breast cancer (TNBC) [116] and HCC [117]. Although combination therapy is associated with a higher incidence of adverse events, the overall safety profile remains manageable for most patients [118].

Thus, ICI–chemotherapy combinations have now become a well‐established standard‐of‐care for numerous malignancies, substantially enhancing both survival rates and patients’ quality of life, which is achieved through modulation of the TME, prevention of treatment resistance, and amplification of therapeutic efficacy. Further efforts should prioritize regimen optimization, toxicity reduction, and the development of personalized treatment approaches guided by biomarker‐driven strategies.

#### 3.2.5 ICIs Combined with Radiation

A growing body of research highlights the promising synergistic antitumor effects of combining ICIs with radiotherapy, mediated through multiple immunomodulatory mechanisms. Radiotherapy enhances tumor immunogenicity by promoting the release of tumor‐associated antigens [119], reshaping the TIME [120], and upregulating immune checkpoints expression [121]. Meanwhile, ICIs can improve vascular normalization, thereby increasing radiosensitivity [122]. Notably, the combination may also trigger the abscopal effect, activating systemic immune responses that control distant, nonirradiated tumor lesions [123].

Clinically, immunotherapy based on ICIs administered sequentially following [124] or concomitant with [125] chemoradiation has been shown to improve PFS and OS in NSCLC. Stereotactic body radiotherapy combined with ICIs also demonstrates promise in advanced NSCLC [126]. For HCC, similar improvements in ORR and PFS, along with favorable safety profiles, have been observed [127]. In melanoma [128] and patients with brain metastases [129], the combined treatment of ICIs and radiotherapy has similarly enhanced systemic immune responses.

Despite this potential, clinical challenges persist, including drug resistance, toxicity management, and patient stratification, future research should focus on optimizing treatment regimens, elucidating underlying mechanisms, evaluating long‐term outcomes, and developing risk mitigation strategies to maximize therapeutic efficacy.

#### 3.2.6 ICIs Combined with Surgery

The application of ICIs has progressively extended from treating advanced malignancies to encompassing peri‐operative management in early‐stage cancers, such as NSCLC, HCC, ESCC, melanoma, RCC, and cervical cancer. ICIs can activate systemic antitumor immunity, enhance the recognition and clearance of micrometastases, and frequently induce substantial tumor shrinkage, thereby facilitating surgical resection. Preoperative ICI treatment also fosters the development of T cell memory responses, which may provide long‐lasting immunosurveillance against residual disease postsurgery [130, 131].

Peri‐operative therapies based on ICIs, either as monotherapy or in combination with chemotherapy, have been shown to increase surgical resection rates and expand surgical opportunities [132, 133]. In NSCLC, for instance, neoadjuvant ICIs used alone [134], in combination with chemotherapy [135], dual immunotherapy [136], or antiangiogenic agents [137], consistently achieve higher rates of major pathological response (MPR) and pathological complete response. Similar results have been reported in HNSCC, where neoadjuvant ICIs combined with chemotherapy or radiotherapy improve disease‐free survival (DFS) rates [138]. For locally advanced gastric cancer, regimens combining ICIs, antiangiogenic agents, and chemotherapy significantly increase the rates of complete pathological response and MPR, with an acceptable safety profile [139]. As adjuvant therapy, ICIs are associated with prolonged DFS and OS, as well as reduced postoperative recurrence [140, 141].

## 4 Current Challenges in Immune Checkpoint Inhibition

Despite the revolutionary progress in cancer immunotherapy, ICIs still confront substantial clinical and translational challenges. Key challenges include accurately identifying patient subgroups most likely to derive benefit, unraveling the mechanistic intricacies of drug resistance, addressing limitations in current efficacy evaluation criteria, and effectively managing irAEs. A thorough comprehension of these obstacles, coupled with the development of tailored solutions, is crucial for optimizing therapeutic outcomes and enhancing long‐term patient benefits.

### 4.1 The Precise Screening of the Beneficiary Population

The effectiveness of ICIs exhibits marked individual variation, driven by intricate spatiotemporal tumor heterogeneity that results in differential responses across metastatic sites and disease stages. This variability highlights the urgent necessity for multidimensional biomarkers and more precise patient stratification strategies.

#### 4.1.1 The Heterogeneity of the Tumor Genome

Tumor genomic characteristics play a pivotal role in determining responsiveness to ICIs. In NSCLC, patients with comutations in NOTCH1 and homologous repair genes [142], as well as those harboring KRAS or TP53 mutations [143], tend to experience enhanced clinical benefits from ICI therapy. In MSS gastrointestinal cancers with high TMB, mutations in CDC73, CTNNA1, and ERBB4 are linked to stronger antitumor immune responses and prolonged survival, whereas alterations in SMAD2, MTOR, or KEAP1 can dampen these responses [144]. Additionally, oncogenic drivers such as EGFR mutations may reduce ICI efficacy [145].

#### 4.1.2 The Heterogeneity of TME

The composition and spatial arrangement of the TME profoundly influence the therapeutic efficacy of ICIs. Tumor‐infiltrating immune cells, including T cells, B cells, and NK cells, serve as indicators of systemic immune competence, with higher baseline lymphocyte counts often predicting better responses to ICIs [146]. In metastatic NSCLC, an immunosuppressive TME may contribute to a lack of response to ICIs [147]. Tumor molecular subtypes also play a role; for instance, MSI‐H colorectal cancers typically respond well to ICIs due to a high neoantigen burden, whereas MSS tumors are often resistant [148].

#### 4.1.3 Host‐Related Factors

Host immunity, gut microbiota composition, and prior treatment history further affect responsiveness to ICIs. Immunosuppressive cells, such as Tregs and MDSCs, can inhibit the activity of effector T cells and reduce the efficacy of PD‐1/PD‐L1 blockade [149]. The gut microbiota influences systemic immunity through the “gut–immune axis,” with taxa such as Bifidobacterium and Akkermansia muciniphila promoting DC maturation and CD8+ T cell expansion [150, 151]. Clinically, a low baseline systemic immune‐inflammation index, such as a neutrophil‐to‐lymphocyte ratio (NLR) of less than 5, is associated with prolonged survival [152], whereas antibiotic‐induced dysbiosis is linked to a reduced ORR [153]. Prior exposure to ICIs may decrease sensitivity to subsequent monotherapy with ICIs but does not preclude the benefits of combination strategies involving ICIs [154]. Notably, current prospective clinical trials of ICIs rarely include patients with complex comorbidities, unique clinical circumstances, or those taking concurrent medications. As a result, the real‐world application of ICIs remains challenging and necessitates careful, individualized management approaches [155].

#### 4.1.4 Heterogeneity of Treatment Regimens

Different treatment regimens, including monotherapy and combination chemotherapy, significantly impact both efficacy and toxicity. In advanced HCC, the triple combination of hepatic arterial infusion chemotherapy with fluorouracil, leucovorin, and oxaliplatin, along with TKIs and ICIs, outperforms dual TKI‐ICI therapy or TKI monotherapy [156]. In NSCLC, chemoimmunotherapy enhances survival compared with ICI monotherapy or other combination regimens [157], although there are notable variations in the incidence of irAEs across different treatment regimens [158].

#### 4.1.5 Limitations of Current Efficacy Biomarkers

Although biomarkers such as PD‐L1 expression, TMB, MSI, and dMMR status guide patient selection, their predictive accuracy remains suboptimal.

First, spatiotemporal heterogeneity undermines the prognostic reliability of these biomarkers. PD‐L1 expression often varies between primary and metastatic sites and can be dynamically altered by prior treatments. Intratumoral heterogeneity and sampling variability further compromise its predictive value [13]. Clinically, inconsistencies are common: some patients with high PD‐L1 expression derive minimal benefit from ICIs [159], while others with PD‐L1‐negative tumors may exhibit robust responses [14]. The predictive power of TMB and MSI/dMMR stems from their association with neoantigen burden. High TMB implies an increased number of tumor‐specific mutations that can generate immunogenic neoantigens, thereby facilitating T cell recognition and antitumor immunity [15]. Similarly, MSI‐H/dMMR tumors produce abundant abnormal peptides that enhance T cell‐mediated cytotoxicity [160]. Nevertheless, a subset of patients with high TMB or MSI‐H/dMMR still demonstrates primary resistance to ICIs [161], highlighting the limitations of relying solely on single biomarkers. These pieces of evidence highlight the limitations of relying on single biomarkers.

Second, standardizing biomarker‐driven prediction faces substantial obstacles. Due to the heterogeneity of detection platforms, patient populations, and samples, the predictive value of PD‐L1 and TMB varies across studies [162].

Third, dynamic changes in PD‐L1 and TMB during treatment may also influence clinical decision‐making [9]. These biomarker fluctuations often result from clonal selection, immune pressure, or tumor remodeling. Given the potential for disease progression or hyperprogression during ICI therapy [163], single‐time‐point biomarker detection offers limited guidance for clinical decisions. However, integrated frameworks that combine genomic, histopathological, and clinical parameters are still lacking. These limitations underscore the urgent need for multidimensional biomarker models to improve patient stratification and optimize ICI‐based therapies.

### 4.2 The Problem of Resistance to ICIs

ICIs resistance is generally categorized as primary or required. Primary resistance, characterized by disease progression within 6 weeks to 6 months after treatment initiation, is frequently associated with low TMB and a lack of PD‐L1 expression [164]. In contrast, acquired resistance emerges after an initial response and is fueled by dynamic adaptations, such as TME remodeling, including the expansion of immunosuppressive cells and T‐cell exhaustion, as well as the loss of tumor antigen expression [165]. The timing of acquired resistance varies based on tumor type and prior therapies.

#### 4.2.1 The Main Mechanisms of Primary Resistance

Primary resistance to ICIs primarily stems from intrinsic immune evasion mechanisms within tumor cells and a highly immunosuppressive TME. Established correlates include low TMB and inadequate or absent PD‐L1 expression. Beyond these factors, multiple pathways converge to weaken the antitumor immune response. These include increased expression of coinhibitory immune checkpoint molecules, such as TIM‐3 and LAG‐3, on tumor cells [166]; aberrant activation of the wingless and int‐1/β‐Catenin signaling pathway, which restricts T cell infiltration [167]; and defects in the antigen presentation machinery [168]. The immunosuppressive TME further exacerbates resistance through the accumulation of Tregs and MDSCs, which secrete cytokines like IL‐10 and TGF‐β to suppress local immunity [169]. At the level of cytotoxic T lymphocytes, impaired responses to IFN signaling due to low IFN alpha receptor 1 expression [170], reduced CD8+ T cell infiltration [171], and T cell exhaustion driven by chronic IFN‐α exposure [172] collectively diminish effector function. Together, these multilayered mechanisms form an integrated network that underpins primary resistance.

#### 4.2.2 The Main Mechanisms of Acquired Resistance

Acquired resistance to ICIs emerges from a complex interplay of tumor cell‐intrinsic adaptations and dynamic host‐tumor interactions. Key mechanisms encompass intrinsic tumor adaptation, microenvironment remodeling, metabolic reprogramming, epigenetic dysregulation, and so on.

Tumor cells evade immune recognition through several critical adaptations. A primary strategy involves reducing immunogenicity, mainly by downregulating MHC molecules [173] and impairing DC function [174]. Furthermore, immune escape is frequently facilitated by activating mutations in oncogenes such as AXL [175], KRAS, serine–threonine kinase 11, and EGFR [176], alongside the inactivation of tumor suppressor genes, notably P53 [177] and phosphatase and tensin homolog [178].

An immunosuppressive TME is pivotal in mediating ICIs resistance. ICIs’ treatment itself can augment the recruitment of Tregs into the TME, fostering an immune‐tolerant environment [179]. This process is exacerbated by other immunosuppressive cells, including MDSCs, TAMs, and cancer‐associated fibroblasts, as well as inhibitory cytokines like TGF‐β, IL‐10, and IL‐35, all contributing to immune evasion and diminishing ICI efficacy [180].

Metabolic competition within the TME also serves as a key resistance mechanism. The rapid proliferation of tumor cells depletes essential nutrients such as glucose, amino acids, and fatty acids, leading to metabolic reprogramming of immune cells. This nutrient scarcity impairs antitumor effector functions and promotes an immunosuppression state [181]. Simultaneously, epigenetic dysregulation, including DNA methylation and histone posttranslational modifications, actively drives immune evasion by disrupting tumor‐associated antigens, chemokines, and genes regulating immune cell activation and tumor recognition [182]. Emerging evidence highlights the correlation between the longitudinal dynamics of the gut microbiota and the development of acquired resistance to ICIs, suggesting potential therapeutic targets to overcome resistance [183].

**FIGURE 4:**
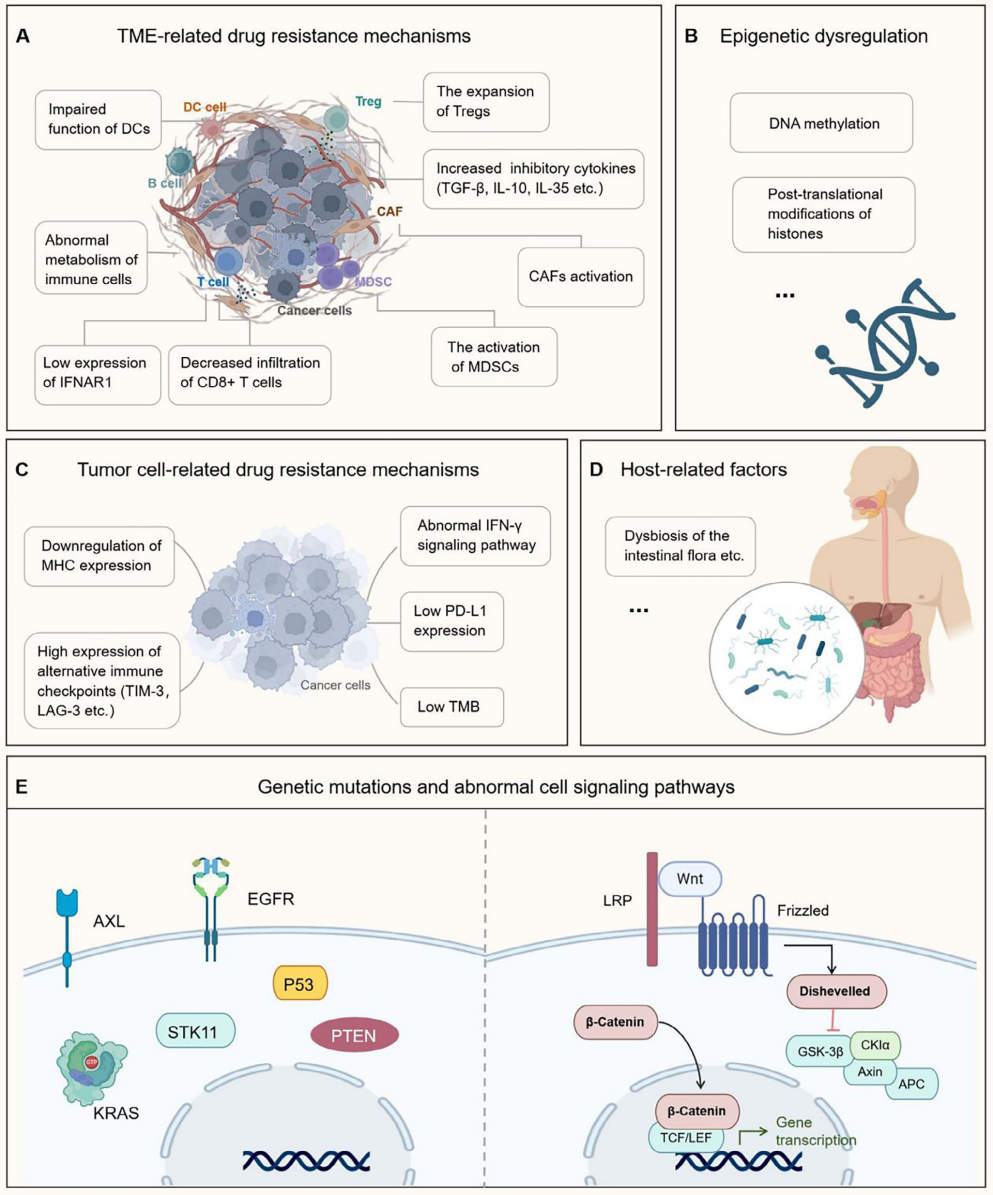
The primary and acquired resistance mechanisms of ICIs. The resistance mechanism of ICIs is complex, involving both external and internal factors of tumor cells. As shown in the figure, panels A, B, and D are external factors of tumor cells, while C and E are internal factors. DC, dendritic cell; Treg, regulatory T cell; TGF‐β, transforming growth factor β; IL, interleukin; CAFs, cancer‐associated fibroblasts; IFNAR1, interferon alpha receptor 1; MDSCs, myeloid‐derived suppressor cells; MHC, major histocompatibility complex; TIM‐3, T cell immunoglobulin and mucin‐domain‐containing‐3; LAG‐3, lymphocyte activation gene 3; IFN‐γ, interferon‐γ; PD‐L1, programmed cell death‐ligand 1; TMB, tumor mutational burden; EGFR, epidermal growth factor receptor; KRAS, Kirsten rat sarcoma viral oncogene homolog; STK11, serine–threonine kinase 11; PTEN, phosphatase and tensin homolog; LRP, lipoprotein receptor‐related protein.

Collectively, these genetic, metabolic, and ecological mechanisms form a highly heterogeneous network of acquired resistance (Figure 4). A deep understanding of these interactions is crucial for developing rational combination therapies that simultaneously target complementary pathways to overcome treatment resistance.

### 4.3 Evaluation of Tumor Response to ICIs

The unique mechanism of action of ICIs gives rise to distinctive tumor response patterns in clinical settings, such as pseudoprogression and hyperprogression. These phenomena pose considerable challenges to conventional response assessment methods, which can be outlined as follows.

#### 4.3.1 Limitations of Response Evaluation Criteria

Atypical response patterns, including pseudoprogression, hyperprogression, and delayed response, significantly challenge conventional efficacy assessment criteria in patients undergoing ICI therapy.

The immune‐related response criteria (irRC), as the inaugural immune‐specific response evaluation framework, enhanced traditional evaluation methods by better capturing these unconventional responses [184]. However, its clinical applicability is constrained by operational complexity, poor reproducibility, a tendency to overinterpret minor lesion alterations, and a potential for overestimating progressive disease. Currently, the combined application of Response Evaluation Criteria in Solid Tumours (RECIST 1.1) and Immune Response Evaluation Criteria in Solid Tumors (iRECIST) remains the primary evaluation standard in both clinical practice and trials, yet it too has shortcomings. For instance, RECIST 1.1 does not recognize pseudoprogression as a valid response [185]. Although iRECIST permits reassessment following initial signs of disease progression, it still inadequately addresses sustained response, rapid progression, and mixed progression patterns, rendering its application somewhat controversial [186, 187].

In 2018, Hodi et al. [188] introduced the innovative imRECIST standard, which distinguishes itself from previous immune‐related efficacy evaluation criteria by focusing on changes in observational metrics and efficacy endpoints via imaging, particularly examining whether PFS translates into OS benefit. The imRECIST standard adopts the single‐diameter measurement approach and selects target lesions and new target lesions in a manner consistent with RECIST 1.1, while aligning with irRC in the management of new lesions. Nevertheless, clinical data supporting imRECIST remain limited.

#### 4.3.2 Limitations of Assessment Methods

Currently, the assessment of ICI responses heavily relies on conventional imaging techniques. Traditional computed tomography (CT) and magnetic resonance imaging primarily gauge tumor size changes and the emergence of new lesions, overlooking shifts in metabolic activity. Although positron emission tomography (PET)/CT holds promise for early metabolic response assessment, its role in the overall efficacy assessment of ICIs remains ambiguous [189]. The potential of more precise radionuclide imaging related to immune cell metabolism also awaits further validation [190]. Moreover, traditional static assessments (e.g., at 6–8 weeks posttreatment) fall short in capturing the dynamic nature of the antitumor immune response. Integrating tumor markers, immune‐related biomarkers from the TME, and host‐derived markers into a dynamic monitoring framework poses significant challenges, with no established strategy currently available.

#### 4.3.3 Limitations of Efficacy Endpoints

Existing research indicates that short‐term efficacy indicators like the ORR do not accurately reflect survival benefits in the immunotherapy landscape. Median survival times, including median PFS or OS, are also flawed as study endpoints due to inherent limitations [188]. Alternatively, 1‐year, 2‐year, or 3‐year absolute survival rates may better delineate survival differences between groups [191]. However, the widespread adoption of these novel methodologies, such as milestone survival analysis, necessitates further validation.

In summary, current strategies for evaluating ICI responses are hampered by limitations in criteria, methods, approaches, and endpoints. Enhanced understanding of ICI mechanisms and metabolic impacts should spur the development of more scientific evaluation methods, novel biomarkers, and updated efficacy standards, ultimately guiding optimized combination therapies and enhancing the efficacy and safety of ICI treatment.

### 4.4 Immune‐Related Adverse Events

**FIGURE 5:**
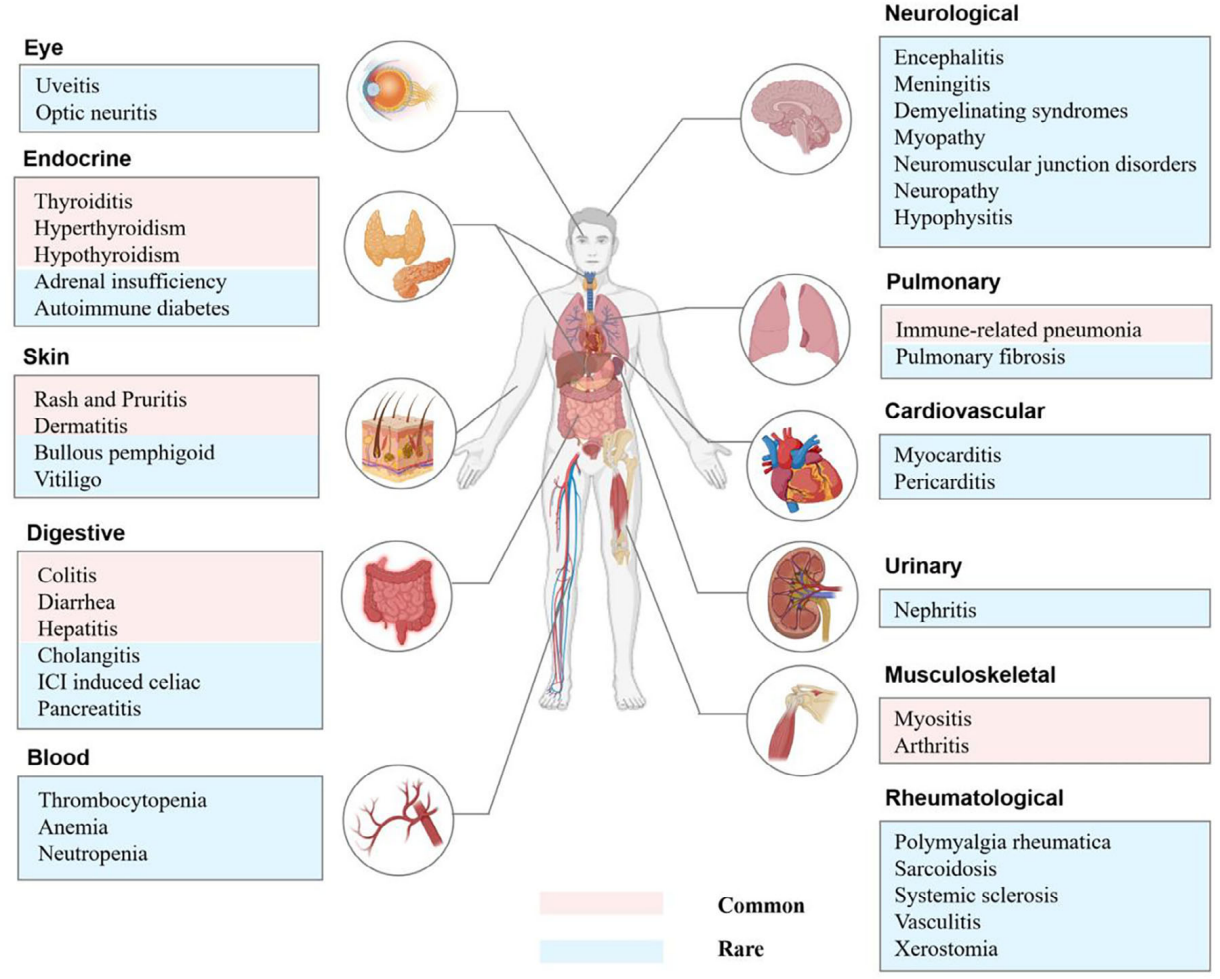
IrAEs based on system classification. IrAEs involve multiple organ systems, mainly including the eye, endocrine system, skin, digestive system, blood system, neurological system, pulmonary system, cardiovascular system, urinary system, musculoskeletal system, and rheumatological disease‐related systems. The pink area represents common irAEs, and the blue area represents rare or uncommon irAEs. ICI, immune checkpoint inhibitor.

irAEs pose a significant clinical challenge in ICI therapy. Their pathogenesis is multifaceted, primarily stemming from the disruption of immune regulatory pathways by ICIs. This enhanced T cell activation, while effective against tumor cells, can also inadvertently target normal tissues, triggering systemic, multisystem inflammatory responses. As illustrated in Figure 5, commonly affected sites encompass the skin, gastrointestinal tract, endocrine system, liver, and lungs, manifesting as dermatitis, colitis, thyroiditis, hepatitis, and pneumonia, among other toxicities. Although many irAEs can be effectively managed with timely intervention, severe or life‐threatening cases may arise, often necessitating treatment interruption or discontinuation [192]. Intriguingly, emerging clinical evidence suggests a link between the occurrence of irAEs and enhanced treatment efficacy, including improved PFS and OS [193]. This paradoxical relationship highlights the intricate balance between antitumor immunity and autoimmunity. Therefore, early identification, individualized management, and multidisciplinary collaboration are crucial for minimizing toxicity while preserving anticancer immune responses. The optimization of irAEs management remains an ongoing pursuit, requiring continuous refinement of clinical protocols and diagnostic strategies.

## 5 Novel Predictive Biomarkers of Response to ICIs

**FIGURE 6:**
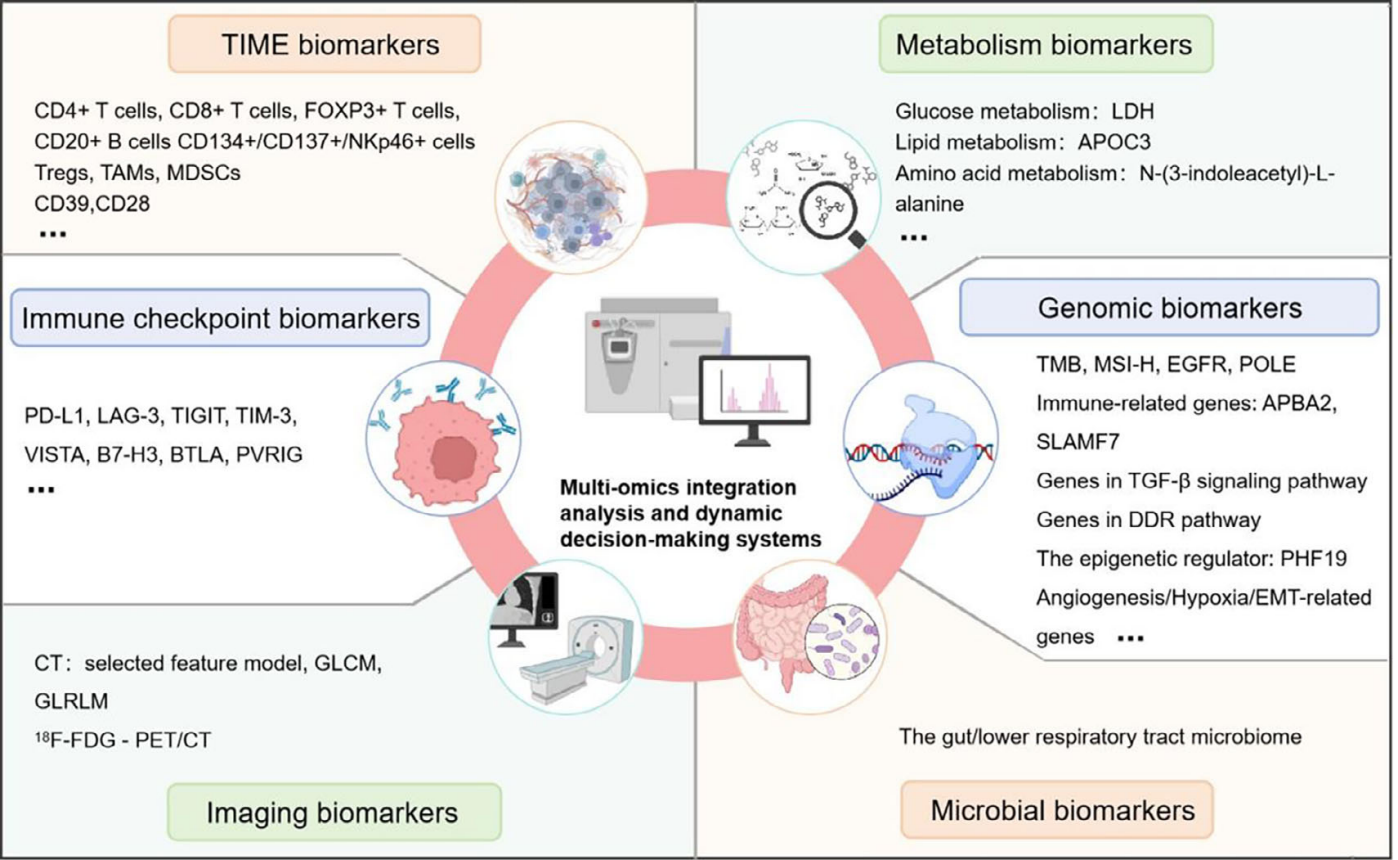
Multidimensional predictive biomarkers of response to ICIs treatment. The therapeutic efficacy of ICIs is comprehensively associated with multidimensional biomarkers, mainly including the following types: biomarkers associated with the TIME, expression levels of immune checkpoint molecules, metabolomic characteristics, genomic biomarkers, microbiomic characteristics, and radiomic characteristics. TIME, tumor immune microenvironment; TAM, tumor‐associated macrophage; MDSCs, myeloid‐derived suppressor cells; LDH, lactate dehydrogenase; PD‐L1, programmed cell death‐ligand 1; LAG‐3, lymphocyte activation gene 3; TIGIT, T cell immunoreceptor with immunoglobulin and tyrosine‐based inhibitory motif (ITIM) domain; TIM‐3, T cell immunoglobulin and mucin‐domain‐containing‐3; VISTA, V‐domain Ig suppressor of T‐cell activation; B7‐H3, B7 homolog 3; BTLA, B and T lymphocyte attenuator; PVRIG, PVR‐related immunoglobulin domain; TMB, tumor mutational burden; EGFR, epidermal growth factor receptor; MSI‐H, high microsatellite instability; POLE, DNA polymerase epsilon; EMT, epithelial–mesenchymal transition; CT, X‐ray computed tomography; ^18^F‐FDG–PET/CT, 2‐deoxy‐2‐[^18^F]fluoro‐d‐glucose (^18^F‐FDG)–positron emission tomography (PET)/CT.

Biomarkers can illuminate tumor immune evasion mechanisms or T cell functional states, thereby predicting response to ICIs. In recent years, with the accumulation of research data, several novel biomarkers have emerged, offering insights into dynamic changes in the TME, immune cell functional status, and tumor cell biological characteristics from multiple perspectives (Figure 6). These advancements hold promise for enhancing patient selection for ICI therapy and uncovering new therapeutic opportunities.

### 5.1 Immune Checkpoint‐Targeted Biomarkers

Novel immune checkpoints like LAG‐3 and TIGIT have been linked to responses to ICIs and show promise as predictive markers. LAG‐3, which dampens T cell activity by binding to MHC class II molecules, has emerged as a crucial therapeutic target. Dual blockade of LAG‐3 and PD‐1 has demonstrated a significant increase in the ORR in NSCLC [194] and extended survival in melanoma patients [195], suggesting that patients with coexpression of LAG‐3 and PD‐L1 may be ideal candidates for combination immunotherapy. Furthermore, the coexpression of multiple inhibitory immune checkpoints, such as PD‐1, TIM‐3, CTLA‐4, and LAG‐3, exacerbates progressive and severe T cell exhaustion [196, 197], ultimately contributing to resistance against ICIs. Other immune checkpoint targets, including VISTA, B7 Homolog 3 (B7‐H3), BTLA, TIGIT, and PVR‐related immunoglobulin domain, have also been confirmed to be involved in the processes of T cell activation, inhibition, and costimulation. These molecules not only represent mechanisms underlying immunosuppression and treatment resistance but also serve as potential novel targets for therapeutic intervention [23].

### 5.2 TIME Biomarkers

The TIME constitutes a complex and heterogeneous ecosystem where various immune cells, stromal elements, and cytokines engage in dynamic interactions with tumor cells, profoundly influencing the response to ICIs.

Early categorizations of TIME were based on the immune contexture, particularly the density and spatial distribution of CD8+ T cells, dividing it into immune‐inflamed, immune‐excluded, and immune‐desert types. Each subtype exhibits a distinct response to ICI treatment [198]. Subsequent studies have highlighted specific immune cell subsets as predictors of ICI efficacy. For example, immune cell types such as CD4+ T cells, CD8+ T cells, transcription factor forkhead box protein 3 (FOXP3)+ T cells, CD20+ B cells, CD134+, CD137+, and NKp46+ cells are positively correlated with OS following ipilimumab treatment [199]. In melanoma, the proportion of memory‐like/exhausted CD8+ T cells [200] and the ratio of CD8+ TCF7+/CD8+ TCF7− T cells [201] are strongly associated with improved response and survival after anti‐PD‐1 therapy. Conversely, immunosuppressive cells like Tregs, TAMs, and MDSCs are commonly linked to resistance or poor response to ICIs across multiple tumor types [120, 202]. Emerging technologies, such as spatial transcriptomics, now allow for the quantification of CD8+ T cell spatial location within the TME. This approach provides a more precise assessment of histological subtypes and immune response status, thereby mitigating the confounding effects of intratumoral complexity on prognostic interpretation [203].

Beyond cellular composition, molecular features on immune or stromal cells also modulate immune phenotype and responsiveness to ICIs. CD39, for instance, is enriched in CD8+ T cells exhibiting characteristics of exhaustion, tumor reactivity, and clonal expansion. Elevated baseline frequencies of CD39+ CD8+ T cells correlate with superior responses to ICIs, and CD39‐related gene signatures may serve as independent predictors of outcomes [204]. Similarly, CD28, which regulates immune function through interaction with CD80/86, represents both a potential biomarker for ICI response and a candidate target for novel immunotherapy designs [205].

### 5.3 Metabolism‐Related Biomarkers

Metabolic reprogramming stands as a hallmark of malignancies, characterized by substantial alterations in glucose, lipid, and amino acid metabolism. These metabolic adaptations not only create an immunosuppressive TME but also influence responses to ICIs by regulating T‐cell function, tumor antigen presentation, and immune checkpoint expression [206]. Clinically, metabolic biomarkers are gaining recognition for their predictive potential. In NSCLC, lower serum levels of the metabolic marker N‐(3‐indoleacetyl)‐l‐alanine are linked to survival benefits following combined treatment with ICIs and chemotherapy [207]. Elevated serum lactate dehydrogenase (LDH) levels, reflecting heightened glycolysis and tumoral hypoxia, consistently correlate with diminished ICI efficacy [208]. In contrast, APOC3, a marker of lipid and cholesterol metabolism, may enhance the immunosuppressive properties of the TME and boost T‐cell function by remodeling high‐density lipoprotein particles. APOC3 levels are inversely associated with tumor burden, and the combination of LDH and APOC3 shows promise as a predictive model for ICIs efficacy [209]. The metabolic enzyme PKM2, which regulates metabolic pathways through the pentose phosphate pathway, represents a potential biomarker for PD‐1 inhibitor efficacy [210]. Overall, metabolism‐related biomarkers provide valuable insights for predicting treatment response and tailoring immunotherapy strategies to individual patients.

### 5.4 Genomic Biomarkers

Significant progress has been made in the development of genomic markers for predicting response to ICIs. In addition to well‐established biomarkers such as TMB, MSI‐H, and driver mutations in EGFR or DNA polymerase epsilon [211], recent studies have uncovered additional gene signatures with clinical significance. In breast cancer, high expression of the immune‐related gene GATA3 is markedly associated with improved recurrence‐free survival rates [212]. In urothelial cell carcinoma, immune‐related genes APBA2 and SLAMF7 are notably correlated with prolonged survival following anti‐PD‐L1 therapy [213]. Conversely, the upregulation of genes involved in angiogenesis [214], hypoxia [215], TGF‐β signaling [216], and epithelial–mesenchymal transition [217] is closely linked to resistance to anti‐PD‐1 therapy. Emerging genomic markers are also attracting attention. For instance, mutations in BRCA1/2, key factors in DNA damage repair, are associated with elevated TMB and enhanced tumor immunogenicity [218]. The epigenetic regulator PHF19 influences immune infiltration and may serve as a prognostic marker in HCC [219]. As research intensifies, these genomic features are expected to provide increasingly refined predictors of ICI response.

### 5.5 Microbial Biomarkers

The gut or lower respiratory tract microbiomes play a pivotal role in shaping responses to ICIs through metabolic production and immunomodulatory pathways. In melanoma, the baseline composition of the gut (fecal) microbiome serves as a predictor of therapeutic outcomes. In particular, patients with microbiomes dominated by Ruminococcaceae exhibit higher response rates to ICIs compared with those dominated by Bacteroidaceae [220]. Similarly, in NSCLC, specific gut microbiome metatranscriptomic signatures correlate with clinical outcomes following ICI treatment [221]. Within the lung, microbial diversity and specific microbiota‐derived metabolites, including those related to lipids and amino acids, remodel the TME through inflammatory cytokine and chemokine signaling, thereby influencing the primary response to ICIs [222]. These findings highlight the potential of microbial biomarkers, whether derived from stool, respiratory samples, or metabolomic profiles, to serve as noninvasive predictors of ICI efficacy.

### 5.6 Imaging Biomarkers

Due to their noninvasive nature and potential for dynamic monitoring, imaging biomarkers have become a focal point in predicting response to ICIs. Radiomic techniques, utilizing X‐ray CT, enable quantitative analysis of tumor phenotypes through tumor segmentation, feature extraction, and model construction. These methods not only reveal macroscopic tumor characteristics but can also predict PD‐L1 expression using a “selected feature model” that includes tumor size, pleural nodularity, pulmonary oligometastases, and the absence of interstitial lung disease, achieving an AUC as high as 0.897 [223]. Three‐dimensional radiomics, employing gray‐level co‐occurrence matrix and gray‐level run‐length matrix analyses, can assess the density of CD8+ T cell infiltration. When integrated with radiological features of PD‐L1 expression, such models demonstrate promise in predicting ICI responsiveness [224]. Concurrently, 2‐deoxy‐2‐[^18^F]fluoro‐d‐glucose (^18^F‐FDG)–PET/CT has gained increasing attention in immuno‐oncology. Standardized uptake values of ^18^F‐FDG correlate with PD‐1/PD‐L1 expression profiles [225], while changes in whole‐body metabolic tumor volume before and after treatment not only aid in assessing long‐term prognosis for patients receiving first‐line combination ICI therapy but may also facilitate early detection of irAEs [226].

Compared with traditional methods like immunohistochemistry, biomarkers derived from microbiome and imaging analyses offer distinct advantages in terms of noninvasiveness, reproducibility, and suitability for serial assessment. Through multiomics integration, dynamic monitoring, and ongoing standardization of acquisition and analytical protocols, these tools are poised to play an increasingly critical role in advancing precision immuno‐oncology.

## 6 Future Directions in ICI Development

The advancement of ICIs is anticipated to unfold through multidimensional innovations. A multiomics‐driven biomarker framework will be pivotal for achieving precise patient stratification. Emerging targets, including TIM‐3 and LAG‐3, offer promising opportunities to expand the scope of immunotherapy. Additionally, the development of bispecific antibodies and novel combination therapies holds potential for overcoming current limitations in treatment efficacy. Meanwhile, tackling various resistance mechanisms, through strategies such as reshaping the TME and enhancing drug delivery technologies, could enhance the durability of therapeutic outcomes. These innovative trends will collectively shape a closed‐loop development model encompassing “precise screening, targeted optimization, and resistance mitigation,” ultimately delivering clinical benefits to a broader patient population.

### 6.1 The Expansion of Therapeutic Indications for ICIs

Supported by a burgeoning array of clinical studies, the development of new agents, and evolving combination strategies, the utilization of ICIs is steadily broadening. Their application is increasingly transitioning from advanced metastatic stages to earlier phases of disease.

#### 6.1.1 Expansion of Cancer Types

Given that immune evasion is a common characteristic of malignant tumors, ICIs are being investigated beyond traditionally responsive cancers such as NSCLC and melanoma. Recent studies have assessed their efficacy in a range of solid tumors, including iCCA, ovarian cancer, and biliary tract cancer. These investigations have uncovered varying efficacy and safety profiles across different tumor types. Both monotherapy and combination regimens involving ICIs have demonstrated promising ORRs and survival trends in these patient populations [227, 228, 229]. As our understanding of the regulatory mechanisms within the TME deepens, expanding the application scenarios of ICIs and identifying potential beneficiary populations will become the primary focus for future clinical indication expansion.

#### 6.1.2 Expansion From Advanced to Early‐Stage Disease

Although ICIs are currently most widely recognized for treating advanced or metastatic cancers, mounting evidence underscores their potential in earlier disease stages. Incorporating ICIs into neoadjuvant and adjuvant treatment regimens marks a promising strategic evolution. For example, in breast cancer, combining ICIs with chemotherapy has demonstrated improved outcomes for patients in early disease stages [230]. Similarly, in NSCLC, immune‐based combinations are increasingly employed preoperatively to enhance resectability and postoperatively to mitigate recurrence risk [135, 231]. Moving forward, ICIs are expected to progressively transition from late‐stage to early‐stage treatment across a broader range of cancers, facilitating more widespread clinical adoption and enhancing survival through stage‐agnostic therapeutic integration.

### 6.2 Optimize Personalized Treatment Strategies of ICIs

ICIs are revolutionizing tumor treatment paradigms. To further enhance therapeutic efficacy, minimize irAEs, overcome resistance, broaden the beneficiary population, and optimize healthcare resource utilization, a steadfast commitment to precision medicine and personalized treatment strategies is imperative. This shift represents not only an inevitable progression in oncology but also a central focus for future research endeavors.

#### 6.2.1 Multidimensional Biomarker Screening

Clinical responses to ICIs exhibit significant heterogeneity [232], largely shaped by tumor genetic characteristics and the immune composition of the TME. Given the intricate and multidimensional regulation of antitumor immunity, reliance on any single biomarker or omics approach falls short of capturing the dynamic interplay of immune checkpoint pathways. Future strategies will necessitate integrative multiomics models that encompass genomic drivers, TME composition, metabolic profiles, and host‐related factors to improve predictive precision and inform rational combination therapy designs.

Accumulating studies highlight the promise of such integration. For example, multimodal models that combine radiological, pathological, and clinical information have refined individualized treatment approaches for HER2‐positive gastric cancer [233]. Integrating normalized bTMB, dynamic monitoring of ctDNA, and initial RECIST responses has effectively predicted sustained clinical benefit in NSCLC, achieving an AUC of 0.878 [234]. The machine learning system SCORPIO leverages blood metabolic features to predict ICI responsiveness across various tumor types [228]. Multiomics platforms further enable real‐time tracking of cell‐free tumor load, assessment of treatment responses, and evaluation of irAE risks [235], while shedding light on complex response patterns such as pseudoprogression and oligoprogression [236].

To sum up, multiomics analytics and dynamic decision‐support systems are transforming ICI evaluation from static patient stratification to real‐time, adaptive precision management. Future efforts should prioritize refining analytical algorithms and developing cost‐effective, noninvasive dynamic monitoring technologies to facilitate personalized immunotherapy management.

#### 6.2.2 Prediction and Precise Management of irAEs

The management of irAEs remains a critical challenge in clinical practice. Regarding predictive biomarkers, an elevated proportion of CD4+ T cells has been linked to anti‐PD‐1‐related pneumonitis in NSCLC [237]. The platelet‐to‐lymphocyte ratio and lymphocyte‐to‐monocyte ratio serve as independent predictors of irAEs [238], while increases in creatine kinase isoenzyme‐MB, cardiac troponin I, and the NLR are associated with ICI‐related myocarditis [239]. Gut microbiota dysbiosis [240] and specific human leukocyte antigen genotypes [241] correlate with severe irAEs and ICI‐related type 1 diabetes, respectively. Radiomics graph models integrating clinical and habitat features can also effectively predict irAEs in lung cancer [242]. Integrating clinical features, peripheral blood biomarkers, and genomic biomarkers into multidimensional predictive models could enhance risk stratification for irAEs. In terms of optimizing management strategies, extracorporeal photopheresis has demonstrated efficacy in treating steroid‐refractory colitis [243], while baricitinib reduces cardiac inflammation and fibrosis by modulating macrophage polarization [244]. Monitoring ICI blood concentrations [245] and metabolic characteristics, such as genetic variations in the vitamin D metabolic pathway [246] and CYP450 polymorphisms [247], can further refine dosing and minimize toxicity.

Overall, irAEs management is evolving from reliance on single biomarkers to integrated, dynamic multiomics approaches. Future research should focus on identifying novel biomarkers, exploring metabolic interventions, and uncovering new therapeutic targets to optimize the risk‐benefit balance of personalized immunotherapy.

### 6.3 Development of Novel Targets and Exploration of Novel Combined Strategies

The field of immune checkpoint inhibition has undergone a significant transformation, shifting from an initial focus on PD‐1 and CTLA‐4 to a new era defined by multitarget synergism. Following PD‐1/CTLA‐4, LAG‐3 has emerged as a key target, with its inhibitor relatlimab, when combined with PD‐1 inhibitors, demonstrating marked improvements in PFS and a favorable safety profile in melanoma, thereby setting a new treatment standard [82]. Other promising targets, such as TIM‐3 [66], TIGIT [248], and VISTA [249], are under active investigation, with antibodies and small molecule agents demonstrating potential to enhance antitumor immunity while minimizing off‐target toxicity.

**FIGURE 7:**
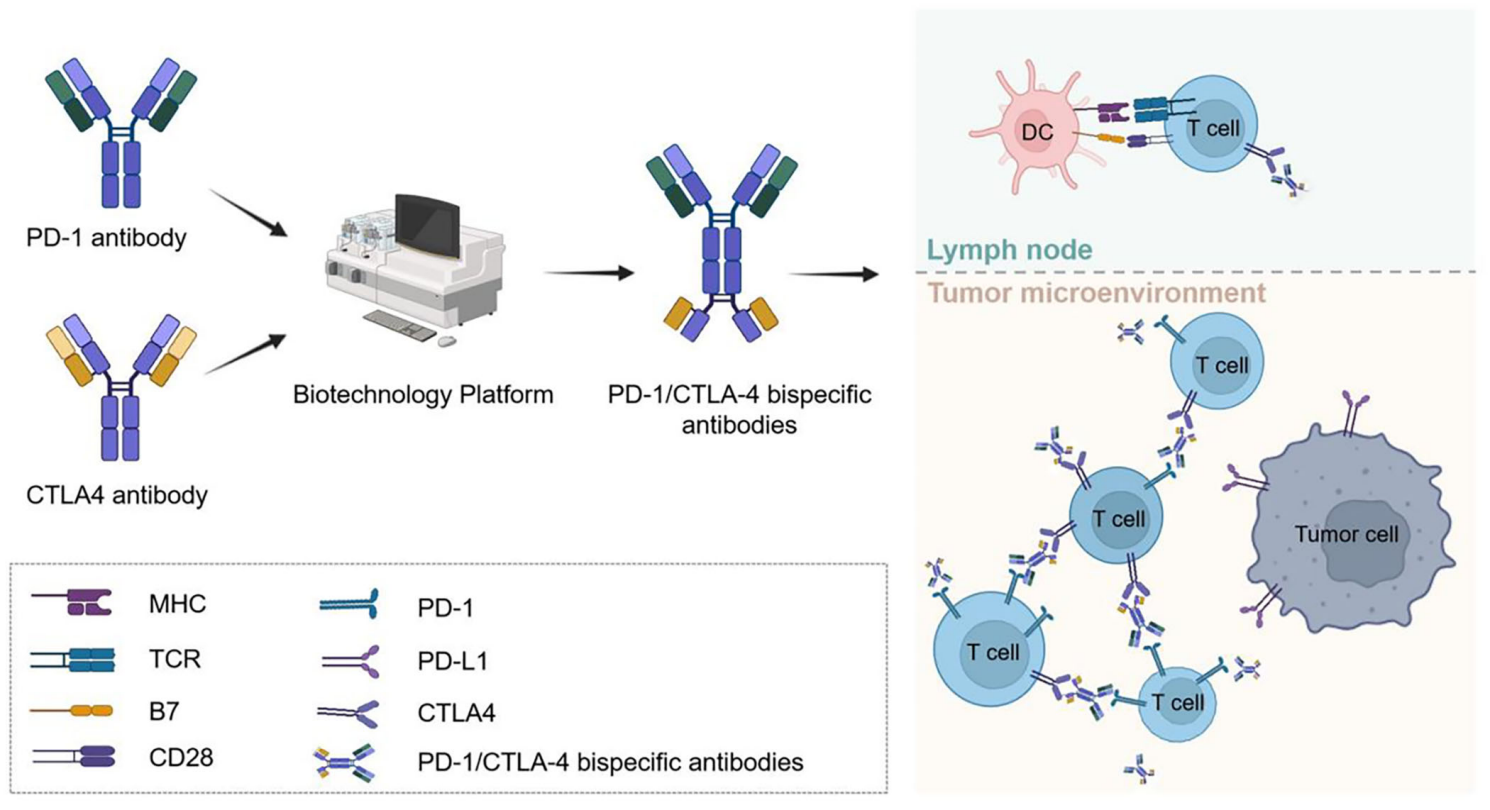
Schematic illustration of the research, development, and mechanism of action of PD‐1/CTLA‐4 bispecific antibodies. The left figure shows the process of generating PD‐1/CTLA‐4 bispecific antibodies through the biotechnology platform by modifying PD‐1 antibodies and CTLA‐4 antibodies. The right figure illustrates the mechanism of action of PD‐1/CTLA‐4 bispecific antibodies. In the lymph node, DCs can interact with T cells through surface molecules to activate T cells. In the TME, PD‐1/CTLA‐4 bispecific antibodies can act on T cells, relieve the inhibition of T cells by tumor cells through the PD‐1/PD‐L1, CTLA‐4 pathways, and enhance the recognition and killing ability of T cells against tumor cells. MHC, major histocompatibility complex; TCR, T cell receptor; PD‐1, programmed cell death 1; PD‐L1, programmed cell death‐ligand 1; CTLA‐4, cytotoxic T‐lymphocyte‐associated antigen‐4; DC, dendritic cell.

Concurrently, innovative biologic formats such as bispecific antibodies (Table 2) and nanobodies are becoming strategic priorities for improving the therapeutic index (Figure 7). For instance, bispecific antibodies targeting both PD‐1 and CTLA‐4 have shown promising activity in extensive‐stage small cell lung cancer [250] and gastric cancer [251], while PD‐1/LAG‐3 bispecific antibodies have demonstrated early signs of efficacy across multiple solid tumors [252].

**TABLE 2:** Key clinical trials assessing bispecific antibodies targeting PD‐1/PD‐L1 signaling.

Targets	Agents	Phase	Cancer types	Current status	Preliminary findings	Trials
PD‐1 × CTLA‐4	QL1706	I	Advanced malignant tumor	Unknown status	RP2D: 5 mg/kg; ORR: 16.9% (overall); 14.0% (NSCLC), 24.5% (NPC), 27.3% (cervical cancer), 7.4% (colorectal cancer), 23.1% (SCLC); median DoR: 11.7 months; TRAEs: 16.0%; irAEs of grade ≥3: 8.1%	NCT04296994 [253] NCT05171790
		II	Stage IIIB/C‐IV NSCLC	Active, not recruiting	Wild‐type EGFR: ORR: 45%; median PFS: 6.8 months; Mutated EGFR: ORR: 54.8%; median PFS: 8.5 months	NCT05329025 [254]
		I/II	Advanced hepatocellular carcinoma	Unknown status	Data not yet available	NCT05603039
		II	Recurrent, or metastatic cervical cancer	Unknown status	ORR: 81%; DCR: 98.3%; median PFS: 14.3 months	NCT05179317 [253]
		II	Extensive‐stage SCLC	Completed	Data not yet available	NCT05309629
	Lorigerlimab (MGD019)	I	Unresectable or metastatic neoplasms	Completed	TRAEs of grade ≥3: 24.2% (total); 28.6% (3 mg/kg); 33.3% (6 mg/kg); 37.5% (10 mg/kg)	NCT03761017
		II	Metastatic castration‐resistant prostate cancer	Active, not recruiting	Data not yet available	NCT05848011
	Cadonilimab (AK104)	Ib/II	Advanced solid tumors	Completed	ORR: 32.3% (cervical cancer); 18.2% (ESCC); 16.7% (hepatocellular carcinoma)	NCT03852251 [255]
		Ib/II	Metastatic or recurrent NSCLC	Completed	ORR: 51.0% (15 mg/kg Q3W); 60.0% (10 mg/kg Q3W); TRAEs of grade ≥3: 59.2% (15 mg/kg Q3W); 25.0% (10 mg/kg Q3W)	NCT04646330 [256]
		Ib/II	Previously treated metastatic NSCLC	Completed	Median OS: 19.6 months (patients who had failed previous platinum‐based doublet chemotherapy and were immunotherapy naïve); 4.9 months (patients who had failed previous platinum‐based doublet chemotherapy and had primary resistance to immunotherapy); 13.2 months (patients who had failed previous platinum‐based doublet chemotherapy and had acquired resistance to immunotherapy)	NCT04172454 [257]
		Ib/II	Advanced HCC	Completed	ORR: 35.5%; median DoR: 13.6 months; median PFS: 8.6; median OS: 27.1 months (6 mg/kg Q2W); ORR: 35.7%; median DoR: 13.7 months; Median PFS: 9.8; median OS: not reached (15 mg/kg Q3W plus plus lenvatinib)	NCT04444167 [258]
		II	Advanced ESCC	Unknown status	Data not yet available	NCT05522894
		II	Extensive stage SCLC	Unknown status	Data not yet available	NCT05901584 [250]
	Vudalimab (XmAb20717)	I	Selected advanced solid tumors	Completed	Data not yet available	NCT03517488
	MEDI5752	I	Advanced renal cell carcinoma	Active, not recruiting	Data not yet available	NCT04522323
		I	Advanced solid tumors	Active, not recruiting	Data not yet available	NCT03530397
PD‐1 × LAG‐3	Tobemstomig (RO7247669)	Ib/II	Melanoma	Completed	RFS: 17.1 months; ORR: 37.5%; AEs of grade ≥3: 12.5%; irAEs of grade ≥3: 2.5%	NCT05116202
		I	Advanced and/or metastatic solid tumors	Active, not recruiting	Data not yet available	NCT04140500
	EMB‐02	I/II	Advanced solid tumors	Terminated	ORR: 6.4%; CBR‐24: 25.5%	NCT04618393 [252]
	Tebotelimab (MGD013)	Ib	Advanced or metastatic solid tumor who failed prior treatment	Terminated	ORR: 5.3% (gastric cancer); 20.0% (TNBC); 8.3% (BTC); 0% (endometrial carcinoma)	NCT04178460 [259]
		I	Unresectable or metastatic neoplasms	Completed	RP2D: 600 mg; TRAEs of grade ≥3: 22.0%	NCT03219268 [260]
PD‐1 × TIM‐3	AZD7789	I/IIa	Advanced solid tumors	Active, not recruiting	Data not yet available	NCT04931654
	Lomvastomig (RO7121661)	I	Advanced and/or metastatic solid tumors	Completed	ORR: 7.9% (metastatic melanoma); 0 (NSCLC); 0 (SCLC); 20.0% (ESCC); DCR: 36.8% (metastatic melanoma); 36.0% (NSCLC); 0 (SCLC); 60.0% (ESCC)	NCT03708328
PD‐1 × TIGIT	Rilvegostomig (AZD2936)	I/II	Advanced or metastatic NSCLC	Recruiting	Data not yet available	NCT04995523
PD‐L1 × CTLA‐4	KN046	II	Advanced NSCLC	Unknown status	ORR: 46.0%; median DoR: 8.1 months; median PFS: 5.8 months; median OS: 26.6 months	NCT04054531 [261]
		II	Advanced solid tumors	Completed	RP2D: 5 mg/kg Q2W; ORR: 12.5%; median DoR: 16.6 months	NCT03733951 [262]
		II	Advanced HCC	Completed	TRAEs of grade ≥3: 47.3%; ORR: 45.5%; median PFS: 11.0 months; median OS: 16.4 months	NCT04542837 [263]
		II	Advanced NSCLC	Terminated	ORR: 13.3% (3 mg/kg); 14.7% (5 mg/kg); median PFS: 3.7 months (3 mg/kg); 3.7 months (5 mg/kg); median OS: 19.7 months (3 mg/kg); 13.0 months (5 mg/kg)	NCT03838848 [264]
PD‐L1 × LAG‐3	FS118	I/II	Advanced malignancies	Terminated	RP2D: 10 mg/kg weekly; DCR: 46.5%	NCT03440437 [265]
	IBI323	I	Advanced malignancies	Unknown status	Data not yet available	NCT04916119
PD‐L1 × TIM‐3	LY3415244	Ia/Ib	Advanced solid tumors	Terminated	DLTs: 0	NCT03752177
PD‐L1 × PD‐1	IBI318 (LY3434172)	Ia/Ib	Advanced malignancies	Completed	RP2D: 300 mg Q2W; TRAEs of grade ≥3: 9.7%; ORR: 15.5%; DCR: 49.5%	NCT03875157 [266]

*Data sources*: ClinicalTrials.gov.

Abbreviations: AEs, adverse events; BTC, biliary tract carcinoma; CBR‐24: clinical benefit rate at 24 weeks; CTLA‐4, cytotoxic‐T‐lymphocyte‐associated antigen 4; DCR: disease control rate; DLTs: dose‐limiting toxicities; DoR, median duration of response; EGFR, epidermal growth factor receptor; ESCC, esophageal squamous cell carcinoma; HCC, hepatocellular carcinoma; irAEs, immune‐related adverse events; LAG‐3, lymphocyte activation gene 3; NPC, nasopharyngeal carcinoma; NSCLC, non‐small cell lung cancer; ORR, objective response rate; OS, overall survival; PD‐1, programmed death 1; PD‐L1, programmed death‐ligand 1; PFS, progression‐free survival; RFS: relapse‐free survival; RP2D, recommended phase 2 dose; SCLC, small cell lung cancer; TIGIT, T cell immunoreceptor with immunoglobulin and immunoreceptor tyrosine‐based inhibitory motif (ITIM) domain; TIM‐3, T cell immunoglobulin and mucin‐domain‐containing‐3; TNBC, triple‐negative breast cancer; TRAEs, treatment‐related adverse events.

The immuno‐oncology arsenal is also expanding with novel combination strategies. Dual immune checkpoint blockade, particularly targeting PD‐1 in combination with LAG‐3 or TIGIT, has become a cornerstone of melanoma therapy [267] and is being effectively extended to other malignancies, including lung cancer, RCC [268], and esophageal/gastroesophageal junction cancer [269]. Moreover, rational combinations of ICIs with small molecule inhibitors, such as IDH1 inhibitors) [270], antibody–drug conjugates (ADCs) [271], or metabolic regulators like statins [272], are yielding compelling synergistic effects. Preclinical evidence suggests that activation of carnitine palmitoyltransferase 1A (CPT1A) can sensitize tumors to ICIs by promoting PD‐L1 degradation [273]. In pancreatic cancer models, a triple therapy combining ICIs with STING/nod‐like receptor protein 3 (NLRP3) agonists and chemotherapy significantly inhibits tumor growth and promotes T cell activation [274]. In addition, in advanced NSCLC, the combination of ICIs and tumor treating fields (TTFields) can reactivate the adaptive immune response and enhance the therapeutic efficacy [275].

These emerging targets and combinatorial approaches are breaking through long‐standing barriers to treatment efficacy, propelling precision immunotherapy into a new dimension.

### 6.4 In‐Depth Analysis of Drug Resistance Mechanisms and Advances in the Intervention Strategies

Recent research has deepened our understanding of the mechanisms underlying resistance to ICIs. A hypoxic TME promotes T cell exhaustion through the HIF‐1α‐ALCAM axis, where the HIF‐1α complex binds to the ALCAM promoter, activating the ALCAM^high^ macrophage‐exhausted T cell axis [276]. Dysregulated tryptophan metabolism due to IDO1 overexpression [277], deficiency of MHC‐I molecules expression (often resulting from B2M mutations) [278], and myeloid cell‐driven immunosuppression through pathways such as the CXCL–CXCR2 axis [279] result in a decrease in tumor antigen presentation capacity, further exacerbating immune escape and promoting ICI resistance.

Beyond traditional combinations with chemotherapy, radiotherapy, or antiangiogenic agents, innovative strategies targeting these resistance mechanisms are rapidly emerging. Fecal microbiota transplantation has been shown to enhance CD8+ T cell function [280] and reduce suppressive cell populations, such as MDSCs and Tregs, by modulating microbial‐derived metabolites like short‐chain fatty acids, potentially reversing ICI resistance [281]. Pharmacologic inhibition of IDO1 with agents such as epacadostat can correct aberrant tryptophan metabolism, facilitating T cell effector function and infiltration [282]. Epigenetic modulators, particularly histone deacetylase inhibitors, can upregulate MHC molecules and tumor antigens, thereby increasing tumor immunogenicity and enhancing T cell recognition [283]. Novel technologies, such as platelet‐derived extracellular vesicles targeting PD‐L1 via the CXCL–CXCR2 axis, have demonstrated significant survival benefits in preclinical models [279]. Collectively, these strategies offer promising avenues for overcoming ICI resistance and refining immunotherapeutic approaches.

## 7 Conclusion and Prospects

ICIs, which target key immune checkpoints like PD‐1/PD‐L1 and CTLA‐4, reverse tumor immune evasion and reshape antitumor immune responses, marking a groundbreaking advancement in oncology. Their efficacy primarily arises from restoring T cell function, enhancing immune cell infiltration, and boosting cytotoxic activity, leading to improved survival in patients with various solid tumors, including melanoma and NSCLC. However, the clinical application of ICIs faces several challenges, such as primary and acquired drug resistance, the management of irAEs, and the limited predictive accuracy of existing biomarkers.

In recent years, significant progress has been made in addressing these obstacles through the discovery of novel therapeutic targets, refined combination strategies, insights into metabolic reprogramming, and innovations in drug delivery technologies. These advancements offer multidimensional solutions to current challenges, with the potential to expand the beneficiary population of ICIs by overcoming resistance, improving patient selection and designing safer, more effective combination regimens.

Moving forward, research efforts should focus on integrating multidimensional technologies. This includes utilizing spatial omics to unravel TME heterogeneity, developing dynamic AI‐driven predictive models, and optimizing interventional strategies through interdisciplinary collaboration. These approaches are crucial for advancing ICIs from a broadly applicable therapeutic option to a more precise and individualized intervention, ultimately revolutionizing the landscape of tumor immunotherapy.

## Author Contributions

All authors conceptualized the manuscript. Hengyi Chen drafted the initial manuscript. Hongling Yang, Lu Guo, and Qingxiang Sun revised the manuscript and provided relevant resources. All authors approved the final manuscript.

## Conflicts of Interest

The authors declare no conflicts of interest.

## Ethics Statement

The authors have nothing to report.

## Data Availability

The authors have nothing to report.
